# Construction of a gene–metabolite–microbiome regulatory network reveals novel therapeutic targets in bladder cancer through multi-omics analysis

**DOI:** 10.1080/07853890.2025.2553220

**Published:** 2025-09-05

**Authors:** Zhiyong Tan, Yinglong Huang, Shi Fu, Haihao Li, Chen Gong, Dihao Lv, Chadanfeng Yang, Jiansong Wang, Mingxia Ding, Haifeng Wang

**Affiliations:** a Department of Urology, The Second Affiliated Hospital of Kunming Medical University, Kunming, Yunnan, People’s Republic of China; b Urological Disease Clinical Medical Center of Yunnan Province, The Second Affiliated Hospital of Kunming Medical University, Kunming, Yunnan, People’s Republic of China; c Scientific and Technological Innovation Team of Basic and Clinical Research of Bladder Cancer in Yunnan Universities, The Second Affiliated Hospital of Kunming Medical University, Kunming, Yunnan, People’s Republic of China

**Keywords:** Bladder cancer, multi-omics, key genes, metabolites, microbiome

## Abstract

**Background:**

Bladder cancer (BLCA) is a prevalent malignancy with substantial consequences for patient health. This study aimed to elucidate the underlying mechanisms of BLCA through integrated multi-omics analysis.

**Methods:**

Tumor and adjacent tissues from BLCA patients underwent transcriptomic, whole-exome sequencing, metabolomic, and intratumoral microbiome analyses. These data were integrated with public datasets to identify key genes, metabolites, and microorganisms. Molecular subtypes were defined by key gene expression and compared for pathways, immune profiles, mutations, immunotherapy response, and drug sensitivity. Prognostic relevance was validated in external cohorts. Single-cell sequencing was applied to reveal cellular localization of key genes.

**Results:**

Three key genes (*AHNAK, CSPG4, NCAM1*), 90 metabolites, and two microbes (*Sphingomonas koreensis, Rhodospirillaceae*) were identified. Key genes negatively correlated with metabolites but not with microbes. BLCA samples were classified into two molecular clusters with distinct ECM organization, metabolic features, immune checkpoint expression, and therapeutic sensitivity. NCAM1 correlated positively with γδ T cells and negatively with M0 macrophages. Single-cell analysis revealed nine major cell types, with fibroblasts displaying the highest expression of key genes, particularly elevated *AHNAK* in specific fibroblast subtypes. Drug prediction and docking identified candidate compounds targeting these genes with stable binding potential.

**Conclusion:**

This comprehensive multi-omics analysis links key genes, metabolites, and microbes to BLCA pathogenesis. Fibroblasts emerge as central regulators, while identified gene–metabolite interactions and microbial associations provide novel insights into tumor heterogeneity. These findings highlight potential biomarkers and therapeutic targets to support precision treatment in BLCA.

## 1. Introduction

Bladder cancer (BLCA) is one of the most common malignancies of the urinary system, and its disease burden has become a significant public health issue. According to the latest epidemiological data, bladder cancer accounts for approximately 573,000 new cases and 212,000 deaths annually worldwide, ranking as the 7th most frequent malignancy in men and the 17th in women. The incidence of bladder cancer increases with age, primarily affecting individuals over 55 years old, with a significantly higher incidence observed in males compared to females [1]. BLCA is classified into non-muscle-invasive bladder cancer (NMIBC) and muscle-invasive bladder cancer (MIBC) based on tumor infiltration depth, which influences clinical diagnosis and treatment strategies. The 5-year survival rates for these subtypes are approximately 90% and 60%, respectively [2]. Currently, the standard treatment for NMIBC is intravesical Bacillus Calmette–Guérin (BCG) immunotherapy, which exerts antitumor effects by activating local immune responses. However, approximately 30–40% of patients develop treatment resistance, highlighting the need to optimize stratified therapeutic strategies based on tumor microenvironment characteristics. Although existing treatment regimens have achieved some success, tumor heterogeneity remains a significant challenge, contributing to drug resistance and recurrence, ultimately affecting patient survival outcomes [3]. In recent years, extensive research has been conducted on the molecular mechanisms of BLCA, encompassing genetic mutations [4], signaling pathways [5], the tumor microenvironment [6], and metabolic pathways [7]. These studies have facilitated the development of refined molecular classifications and prognostic models based on gene and protein expression profiles. Nevertheless, the multifactorial nature of tumorigenesis-including genetic mutations, metabolic reprogramming, and microbial interaction-remains incompletely understood. Therefore, comprehensive multi-omics analysis to better understand the disease’s multilevel heterogeneity is essential for improving patient prognosis and remains a critical area of investigation.

Single-cell transcriptomics has become a key method in developing predictive models for the survival of patients with BLCA and immune microenvironment profiling [8–10]. While this approach has revealed some gene expression variability, it has not fully addressed inter-patient and intra-tumor heterogeneity. Advancements in scientific techniques, particularly whole-exome sequencing (WES) and metabolomics, have been increasingly employed in tumor research. WES data suggest that normal uroepithelial cells can accumulate between 500 and 2,000 mutations by the ages of 50–65, with BLCA exhibiting a higher tumor mutational burden (TMB), primarily driven by APOBEC3 cytidine deaminase activation [11]. Furthermore, variations in glucose, lipid, and carnitine metabolism across different grades of BLCA have been associated with tumor invasion and metastasis [11], indicating that genetic mutations and metabolic pathways are key contributors to BLCA pathogenesis. The microbiome may play a causative or cofactor role in genitourinary malignancies [12]. For instance, Sun et al. observed greater microbial diversity in NMIBC compared to MIBC, with distinct microbial species dominating in each subtype, as revealed by 2bRAD sequencing of the microbiome [13]. Increased microbial abundance has been linked to higher recurrence and progression risks in tumor individuals compared to healthy controls [14]. Although previous multi-omics studies [15] have analyzed gene–metabolite associations in BLCA, they lack integration of the microbiome and single-cell dimensions. Therefore, this study aims to elucidate how distinct molecular features collectively influence disease initiation, progression, and therapeutic response by integrating transcriptomics, WES, metabolomics, intratumoral microbial community profiling, and single-cell data, thereby providing a comprehensive understanding of the biological system.

In this study, transcriptomics, WES, metabolomics, intratumoral microbiome analysis, and single-cell profiling were integrated to identify the key genes, metabolites, and microorganisms associated with BLCA. Additionally, their functions and regulatory interactions were systematically investigated across multiple biological hierarchies. Further, this study classified BLCA based on molecular profiling of key genes and explored the underlying molecular mechanisms in detail. Finally, the expression patterns of these key genes were examined within the context of specific cell types at single-cell resolution. These findings offer new insights into the molecular mechanisms driving BLCA and hold promise for developing more targeted therapeutic strategies, thereby advancing personalized treatment approaches for patients with BLCA. The flowchart of this study is shown in Figure S1.

## 2. Materials and methods

### 2.1. Patient selection and sample collection

A total of 35 cancer and adjacent normal tissue samples (Table S1) were collected from patients diagnosed with BLCA between March 2024 and December 2024 for comprehensive sequencing, including transcriptome, WES, metabolome, and intratumoral microbiome analysis. To minimize the risk of endogenous contamination during surgery, all samples were aseptically collected in the operating room, with clean tissue blocks selected whenever possible. The matched adjacent normal tissues were collected at least 3 cm away from the visible tumor margin. The samples were then cryopreserved at −80 °C for storage. Prior to experimentation, both tumor and adjacent normal tissues underwent hematoxylin–eosin (H&E) staining and immunohistochemical (IHC) analysis to verify their histological normality and cellular proliferation status. All patients were treated at The Second Affiliated Hospital of Kunming Medical University, and the study received approval from its Research Ethics Committee. Written informed consent was obtained from all participants.

### 2.2. Data source

The Cancer Genome Atlas (TCGA)–BLCA dataset was sourced from the University of California Santa Cruz (UCSC) Xena database (https://xena.ucsc.edu/) and served as the transcriptome training set [8]. This dataset included RNA sequencing data from 411 BLCA tumor samples and 9 control tissue samples, along with corresponding clinical and survival data. Additionally, the GSE13507 dataset (platform: GPL6102) was acquired from the Gene Expression Omnibus (GEO, https://www.ncbi.nlm.nih.gov/geo/) as an external validation set for transcriptome analysis [16]. It contains 188 BLCA tumor tissue samples and 68 control bladder mucosa tissue samples. Furthermore, two BLCA-associated single-cell datasets were incorporated into the study: GSE135337 (platform: GPL24676) and GSE222315 (platform: GPL24676), both obtained from the GEO database. The GSE135337 dataset includes seven BLCA tumor samples and one paraneoplastic tissue sample, while the GSE222315 dataset includes nine BLCA tumor samples and four paraneoplastic tissue samples.

### 2.3. Analysis of transcriptome sequencing data

To enhance data quality and standardization, raw reads from the transcriptome sequencing data of 35 BLCA tumor and paraneoplastic samples were first subjected to quality control using FastQC and FastP. Low-quality reads were removed to obtain clean data. The clean data were then aligned to the human genome (GRCh38) using TopHat 2.0. Transcripts per kilobase of exon model per million mapped reads (TPM) values were calculated for each gene to evaluate mRNA distribution bias. Principal component analysis (PCA) was performed on BLCA tumor and paraneoplastic samples using the ‘ropls’ R package (version 1.34.0) [17] to assess data variability. Additionally, Spearman correlation coefficient (Cor) and its significance were calculated using the ‘cor’ function (version 0.92) [18] to assess the correlation between the two groups of samples (|Cor| > 0.3, *p* < 0.05). Differential expression analysis was performed to identify differentially expressed genes (DEGs) between the BLCA tumor and paraneoplastic sample groups. The R package ‘DESeq2’ (version 1.42.0) [19] was utilized to determine differential gene expression, with adjusted *p* values <0.05 and |log_2_ fold change (FC)| > 0.5 [20]. To elucidate the biological functions and pathways associated with DEGs, the Gene Ontology (GO) and Kyoto Encyclopedia of Genes and Genomes (KEGG) enrichment analyses were performed on DEGs using the ‘clusterProfiler’ package (version 4.7.1.3) [21]. Significantly enriched terms/pathways were identified with an adjusted *p* values <0.05.

### 2.4. Analysis of WES data

To identify significant mutant genes (SMGs) in both sample groups, tumor mutation data from 35 BLCA tumor samples were analyzed using the ‘maftools’ package (version 2.17.10) [22]. The oncodrive function was used to calculate whether the mutation frequency of genes was significantly higher than the random background. The core parameter was set to minMut = 1 to retain genes with at least one mutation and exclude those without mutations. Additionally, the *Z* test (pvalMethod = ‘zscore’) was used to calculate the *Z* score of the mutation frequency to assess the deviation from the expected background. Finally, multiple testing correction was performed using the false discovery rate (FDR) to control for false positive results, and genes with an FDR < 0.05 were selected as significant mutant genes. GO and KEGG enrichment analyses of the SMGs were also performed using the same methods with a significance threshold of *p* < 0.05 to explore the biological pathways associated with these genes. The Oncobox Pathway Database (OncoboxPD, https://open.oncobox.com), which comprises 51,627 human molecular pathways, was used to process the data uniformly. KEGG pathway enrichment analysis was conducted for the SMGs using OncoboxPD with an adjusted *p* value <0.05, and pathway activation levels (PALs) were calculated for all KEGG pathways to assess the activation and inhibition status of the pathways enriched by SMGs.

### 2.5. Analysis of metabolomic

To assess metabolomic variations between the two sample groups, PCA was performed on the metabolomic sequencing data. The correlation between the two groups was evaluated using Spearman analysis, with a correlation coefficient |Cor| > 0.3 and *p* < 0.05. Differentially expressed metabolites (DEMs) between cancer and normal samples were identified based on the variable importance in projection (VIP) values derived from orthogonal partial least squares discriminant analysis (OPLS-DA) and the *p* values from paired Student’s *t* tests. Thresholds for identifying DEMs were set to VIP > 1 and adjusted *p* value <0.05. To investigate the biological processes associated with the DEMs, a metabolic profile was generated using MetaboAnalyst. Compound names of DEMs were converted to KEGG IDs, and pathway analysis was performed using the KEGG database with a significance threshold of *p* < 0.05.

### 2.6. Analysis of intratumoral microbial data

To ensure the accuracy of subsequent analyses, the rarefy function was first used to rarefy the original OTU counts, adjusting the sequencing depth of all samples to a specified threshold. Next, FASTP was employed for data quality control to remove low-quality sequences, and the UCHIME algorithm was used to eliminate chimeric sequences. Sequences with greater than 97% similarity were clustered into the same operational taxonomic unit (OTU) using UPARSE (http://drive5.com/uparse/). Taxonomic information for these clustered OTUs was annotated and evaluated using the Mothur algorithm and the Silva database (https://www.arb-silva.de/). Additionally, alpha and beta diversity analyses were performed to assess and compare the composition of intratumoral microorganisms between cancer and normal groups. For alpha diversity analysis, the vegan (version 2.6-10) package [23] was used to calculate diversity indices including Shannon, Simpson, InvSimpson, Pielou, ACE, and Chao1, so as to quantify the richness and evenness of microbial communities within samples. The non-parametric Wilcoxon rank-sum test was employed to compare the significance of differences in each alpha diversity index between the tumor group and normal control group. For beta diversity analysis, the Microeco (version 1.8.0) package [24] was used to perform Principal Coordinate Analysis (PCoA) and Non-metric Multidimensional Scaling (NMDS). Bray–Curtis distance, Jaccard distance, and weighted/unweighted UniFrac distances were selected to quantitatively evaluate the similarity and difference of microbial community composition among samples. Among them, the phylogenetic tree was generated by the rtree function of the ape (version 5.8-1) package [25], which was used to visualize the evolutionary relationships of microbial taxa. Bar charts were created to illustrate the relative abundance of intratumoral microorganisms at the genus levels for both sample groups. To further investigate the differential microbiota in samples from different groups, the LEfSe algorithm was employed. At the genus level, the Kruskal–Wallis test was combined to analyze the differences between tumor and normal groups. Linear Discriminant Analysis (LDA) Effect Size (LEfSe) was then applied, with a threshold of LDA > 2 and *p* < 0.05 to identify specific microbial taxa between the two groups. To further explore the biological processes associated with these differential microorganisms, KEGG pathway enrichment analysis was conducted using PICRUSt2 (version 2.6.2) software [26].

### 2.7. Co-analysis of transcriptome and WES

To identify genes that exhibited significant differences at both the transcriptomic and WES levels, DEGs derived from transcriptome analysis were merged with SMGs from WES data. The resulting genes were considered candidate genes for further analysis. These candidate genes underwent GO and KEGG enrichment analyses, with an adjusted *p* value threshold of <0.05. To further investigate the prognostic value of the candidate genes, the optimal expression thresholds were calculated according to the surv_cutpoint function in the ‘survminer’ package (version 0.4.9) [20] (the gene expression cut-off points that could maximize the discrimination of the prognostic differences among patients were calculated based on the maximal selected rank statistic algorithm), and the patients with BLCA in the TCGA–BLCA dataset were divided into high and low expression sets. Survival analysis was then performed using the ‘survminer’ package (version 0.4.9) [27], and Kaplan–Meier (KM) survival curves were constructed to evaluate the overall survival (OS) of patients in these two groups. Candidate genes exhibiting significant OS differences (*p* < 0.05) between the groups were considered to have prognostic value and were defined as candidate prognostic genes. In addition, to assess the stability and reliability of the prognostic gene expression patterns and prognostic value, expression analysis and receiver operating characteristic (ROC) analysis were performed at the transcriptomic level, TCGA–BLCA and GSE13507 datasets, respectively. The Wilcoxon rank-sum test was used to compare the expression differences between the cancer and normal groups (*p* < 0.05). ROC curves were plotted using the ‘pROC’ (version 1.18.4) [26] R package to assess the ability of candidate prognostic genes to differentiate between cancer and normal samples. Genes with an area under the curve (AUC) > 0.7 across all three transcriptomic datasets were considered as biomarkers for further study.

### 2.8. Combined analysis of transcriptome, metabolome, and intratumoral microbiome

To comprehensively analyze the pathogenesis and progression of bladder cancer, the transcriptome, WES and metabolomic, and tumor-associated microbiome data were systematically integrated for comprehensive analysis. First, biomarkers selected from the transcriptomic and WES data were subjected to Spearman analysis with DEMs from the metabolomic data (|Cor| > 0.3 and *p* < 0.05), identifying key gene 1 and key metabolite 1 that were significantly correlated at both the transcriptomic and metabolomic levels. The potential interactions were further investigated. Subsequently, using the same method parameters, biomarkers were correlated with the differentially abundant microbiota in the tumor-associated microbiome data, successfully identifying key gene 2 and key microbiome 1. Similarly, the correlation analysis of DEM and differential microbes was performed to obtain key metabolite 2 and key microbiome 2. Subsequently, the ‘VennDiagram’ package was used to perform intersection analysis on key gene 1 and key gene 2, key metabolite 1 and key metabolite 2, and key microorganism 1 and key microorganism 2, in order to obtain the final key genes, key metabolites and key microorganisms. Spearman correlation analysis was then conducted to assess the relationships between these key factors.

### 2.9. Analysis of BLCA clusters

To identify molecular clusters of BLCA associated with key genes, a consensus clustering was performed on BLCA samples in the transcriptome sequencing data based on key genes, using the ‘ConsensusClusterPlus’ package (version 1.65.0) [28]. The parameters were set with maxK = 7, reps = 100, pItem = 0.95. The appropriate number of clusters (K) was determined by analyzing the cumulative distribution function curve in combination with the ‘fpc’ (version 2.2-12) package [29]. Ultimately, the BLCA samples were classified into different clusters. The distribution of these clusters was further assessed and visualized using PCA and *t*-distributed stochastic neighbor embedding (tSNE), and UMAP. Inter-cluster differences were then examined by analyzing DEGs with criteria of |log_2_FC| > 0.5 and *p* < 0.05, which allowed for the identification of DEGs between clusters. GO and KEGG enrichment analyses were subsequently performed on the inter-cluster DEGs, with an adjusted *p* value threshold of <0.05. To investigate the association of microbial communities in different clusters, association networks between microbial communities (at the genus level) were constructed using intratumoral microbiome sequencing data. Functional predictions of different clusters were also conducted to explore functional differences. Similarly, inter-cluster DEMs were identified from metabolome sequencing data, using VIP > 1.00 and *p* < 0.05 as screening thresholds. The metabolic pathways associated with the transformed inter-cluster DEMs were analyzed using the KEGG database (*p* < 0.05). Further in-depth analyses were conducted to investigate the biological pathways and metabolic processes specific to different BLCA clusters. Gene Set Enrichment Analysis (GSEA) using the ‘clusterProfiler’ package (version 4.7.1.3) [21], the HALLMARK gene set (h.all.v2023.1.Hs.symbols.gmt) as the reference exploring the pathways or functions that were significantly enriched among different subtypes. Furthermore, using metabolism-related pathways [30] as the reference gene set, the ‘GSVA’ package was employed to evaluate the pathway scores of all samples through the single-sample gene set enrichment analysis (ssGSEA) algorithm, thereby comparing differences in metastasis- and metabolism-related pathways between subtypes (*p* < 0.05).

To explore the differences in the tumor immune microenvironment (TIME) between subtypes, the CIBERSORT tool was utilized to assess the abundance of 22 immune-infiltrating cell types in all samples. Subsequently, Wilcoxon rank-sum tests were performed to compare differences in immune-infiltrating cells between subtypes (*p* < 0.05). To further uncover potential associations between differential immune cells and key genes/microbes, the ‘cor’ function was employed to conduct Spearman correlation analyses to evaluate correlations between key genes, key microbes, and immune cells, respectively. Additionally, inter-cluster differences in 66 immune checkpoint genes [31], immune-circulatory pathway activity, patient responses to immunotherapy, somatic mutations, mutations in typical oncogenic pathways, and drug were deeply investigated (Wilcoxon rank-sum test, *p* < 0.05). To compare genetic mutation profiles between subtypes, the ‘Maftools’ R package was used to analyze somatic mutation data including single nucleotide variants (SNV) and single-nucleotide polymorphisms (SNP), and Fisher’s exact tests were applied to compare mutation frequency differences of high-frequency mutated genes between subtypes (*p* < 0.05). Wilcoxon rank-sum tests were also used to compare TMB differences between subtypes (*p* < 0.05). To explore the potential roles of key microorganisms in immune-related processes, the correlation between key microorganisms and major histocompatibility complex (MHC) molecules, immune activators, and immunosuppressants was assessed using Spearman analysis on intratumoral microbiome sequencing data. Expression levels of key genes, key metabolites, and key microorganisms across different clusters were compared using the Wilcoxon rank-sum test in transcriptomic, metabolomic, and microbiome sequencing data, respectively, with a significance threshold of *p* < 0.05. The key genes that exhibited significant expression differences across BLCA clusters were selected for subsequent analysis.

### 2.10. Association analysis of clinical characteristics of key genes

To assess the expression of key genes in relation to clinical characteristics, patients with BLCA from the TCGA dataset were stratified into distinct clinical subgroups based on varying clinical features (T/M/N staging, age, gender, Stage). Differential expression of key genes across these subgroups was evaluated using the Wilcoxon rank-sum test, with a significance threshold of *p* < 0.05. Additionally, correlations between key genes and individual clinical characteristics were analyzed using Spearman correlation. For further stratification, patients with BLCA were classified into two groups based on the median expression level of each key gene. KM survival curves were then constructed to evaluate the OS of patients with high and low expression levels of each key gene within the different clinical subgroups.

### 2.11. Enrichment analysis of key genes

To investigate the biological pathways associated with the key genes in BLCA, single-gene GSEA was performed based on transcriptome sequencing data. This analysis utilized the ‘c2.cp.kegg.v7.0.symbols.gmt’ gene set as a reference for the transcriptome sequencing data, with an adjusted *p* value threshold of <0.05. Spearman correlation coefficients between each core gene and all other genes were first calculated, and then all genes were ranked by the correlation coefficients from highest to lowest to construct a list of related genes for each core gene. The ‘clusterProfiler’ package (version 4.7.1.3) [21] was then used to perform GSEA on the genes in the list to reveal the potential biological functions of the core genes in the disease. In the TCGA dataset, patients with BLCA with survival information were grouped based on the median expression value of each key gene. GSEA was used to explore significantly enriched pathways or functions between the high and low expression subgroups of each key gene, using the ‘HALLMARKgenesets: h.all.v2023.1.Hs.symbols.gmt’ reference gene set (adjusted *p* value <0.05).

### 2.12. Drug prediction and molecular docking

To identify potential therapeutic agents for BLCA, molecular compounds or agents interacting with the key genes were predicted using the L1000 FWD platform (https://maayanlab.cloud/l1000fwd/). The thresholds were set as adjusted *p* value <0.05, odds ratio >1, and Combined Score >1000. The drugs were ranked in descending order of Combined Score, and the top-ranked drugs that were negatively correlated with the key gene were selected as ligands for molecular docking. The molecular structures of the drugs and the three-dimensional structures of the proteins were then downloaded from the Pubchem (https://pubchem.ncbi.nlm.nih.gov/) and PDB (http://www.rcsb.org/) databases. Subsequently, the Surflex–Dock algorithm in Sybyl-X (version 2.0) software [32] was used to perform molecular docking between core genes and corresponding drugs. The ligand flexibility rotation parameter was set to simulate the real binding conformation, and the receptor binding pocket radius was set to 10 Å to cover potential binding sites. The energy score threshold was set to −5 kcal/mol to select candidate drugs with stable binding ability. Hydrogen bonds and hydrophobic interactions were visualized by PyMOL (version 2.5.4) software.

### 2.13. Information, function, and expression analysis of proteins encoded by key genes

Information on key genes, their encoded proteins, and related functions was further investigated using the National Center for Biotechnology Information (NCBI) database. Additionally, the expression of key genes in pan-cancer was analyzed by comparing gene expression levels in pan-cancer tumors versus control samples from the TCGA database. The expression of these key genes in different tissues and at the protein level in both tumor and normal samples was further investigated using The Human Protein Atlas (HPA, https://www.proteinatlas.org/).

### 2.14. Analysis of single-cell data

To ensure the accuracy of the analysis, low-quality cells were first filtered out, the GSE135337 and GSE222315 single-cell datasets were first integrated using the IntegrateLayers function in the Seurat package (version 4.4.0) [33] with canonical correlation analysis (CCA) to correct batch effects, followed by quality control of the single-cell RNA sequencing data. Filtering criteria were set as nCount < 20000, 100 < nFeature < 5000, and percent.mt < 10%. Meanwhile, genes with fewer than 200 genes per cell or covered by fewer than 3 cells were removed to ensure the quality of analyzed cells and exclude low-quality cells. After passing the quality control, the LogNormalize method was applied to standardize the filtered data to eliminate the impact of sequencing depth and other experimental conditions. Subsequently, the FindVariableFeatures function combined with the vst method was utilized to screen the top 2000 highly variable genes. Principal component analysis (PCA) was then performed on the data, and the JackStrawPlot function was used to evaluate and determine the top 30 principal components for subsequent analysis. Based on this, the FindNeighbors and FindClusters functions (resolution = 1) were employed to identify small cell clusters, and UMAP was used for nonlinear dimensionality reduction clustering to clearly display the distribution of cell clusters. To deeply analyze cell types, the FindAllMarkers function was used to identify marker genes in each cell cluster (|log2FC| > 1.0, adj. *p* < 0.05) [34,35], and heatmaps were drawn to visually display the expression pattern of these marker genes in different cell types. Meanwhile, SingleR algorithm was used to assist in annotating identified cell types. The expression of each key gene in the different cell types was then assessed and visualized in the single-cell dataset. Differences in gene expression levels between cancer and control samples were compared.

The ReactomePA package (v 1.34.0) was used to analyzed the KEGG functional enrichment of all cell types (*p* < 0.05). Based on the differential expression of key genes in each cell type, the functional enrichment results, and relevant literature, key cells were determined. Ligand–receptor interactions between the key cells and other cell types were analyzed to explore cell communication. To better understand the heterogeneity of the key cells, dimensionality reduction clustering was applied, and the key cells were further reclustered into subclusters. These subclusters were annotated using reference sources [36–38] to identify the key cell clusters. The potential differentiation trajectories of these key cell clusters were then explored through mimetic temporal sequencing analysis, and the expression of key genes during different temporal sequences was visualized. Additionally, upstream regulators of key genes in the various clusters of key cells were identified to speculate on the mechanisms of action of the key genes.

To further examine the degree of malignancy in the cell types, epithelial cells identified in the previous analyses were reclustered into subclusters. The clusters of these epithelial subclusters were determined, and temporal analysis of the epithelial cell clusters was performed to examine their differentiation trajectories according to malignancy degree. The expression of key genes in different time series was visualized, and comparisons of gene expression across cell clusters with varying degrees of malignancy were made.

### 2.15. Statistical analysis

Bioinformatics analyses were performed using the R programming language (version 4.2.2). First, PCA was conducted using the procmp function in the R package ‘ropls’ (version 1.34.0) to evaluate the distribution characteristics among samples. Then, Spearman correlation analysis was performed using the ‘cor’ function (version 0.92) to assess the correlation between the two groups of samples. Differential gene expression analysis was carried out using the ‘DESeq2’ package (version 1.42.0) to screen for differences in gene expression levels between BLCA tumor and normal control samples. Mutation information was analyzed using the ‘Maftools’ package (version 2.17.10). Amplifications and deletions of copy number variation (CNVs) were analyzed using the Gistic2.0 tool. To screen for differential metabolites, Student’s *t* test was employed. Meanwhile, LEfSe was used to identify specific microbial taxonomic differences between the two groups of samples. Functional enrichment analysis was performed using the ‘clusterProfiler’ package (version 4.7.1.3). KM survival curves were plotted using the R package ‘survminer’ (version 0.4.9) to compare the OS of candidate genes across different expression groups. ROC curves were drawn using the R package ‘pROC’ (version 1.18.4) to evaluate the predictive performance of candidate genes. Additionally, Cytoscape software was used to construct interaction networks of samples, and consensus clustering analysis of BLCA samples was performed using the ‘ConsensusClusterPlus’ package (version 1.65.0). GSVA was conducted using the ‘GSVA’ package (version 1.49.4). Immunoinfiltration analysis was performed using CIBERSORT to assess the abundance of 22 immune-infiltrating cell types. Fisher’s test was used to compare mutation frequency differences of high-frequency mutated genes across subtypes. Single-cell analysis was performed using the ‘Seurat’ package (version 4.4.0), with batch correction conducted using CCAIntegration. The Wilcoxon rank-sum test was used to compare differences between two groups. All statistical analyses were set with a significance threshold of *p* value <0.05.

## 3. Results

### 3.1. Quality assessment of sequencing data

To assess the quality of the transcriptome sequencing data, gene expression profiles were analyzed. Box plot analysis of the gene expression level distribution (TPM values) of the samples, it was found that the gene expression levels of cancer and paracancerous tissue samples from four BLCA patients (36, 37, 38, and 39) significantly deviated from the overall distribution, which were excluded. The remaining 70 samples, comprising 35 tumor and 35 paraneoplastic samples, were retained for further analysis (Figure S2a). PCA of these samples demonstrated distinct clustering within each group (Figure S2b), and correlation analysis showed a generally positive correlation between samples from the two groups (Figure S2c). Metabolome sequencing data effectively distinguished tumor from normal samples (Figure S2d). Intratumoral microbiome sequencing data were also analyzed to identify cluster OTUs. Rarefaction curves reached a plateau, indicating sufficient sequencing depth (Figure S2e). Rank–abundance curves illustrated the richness and evenness of the samples (Figure S2f). The microbial community in the tumor samples had a higher proportion of dominant species and lower evenness, while the species abundance distribution in the normal samples was more balanced. The species cumulative box plot revealed that species diversity increased with sample size until stabilizing (Figure S2g). These results collectively demonstrate high sequencing quality and effective differentiation of the samples in this study.

### 3.2. Identification and function of DEGs, SMGs in BLCA

**Figure 1.**
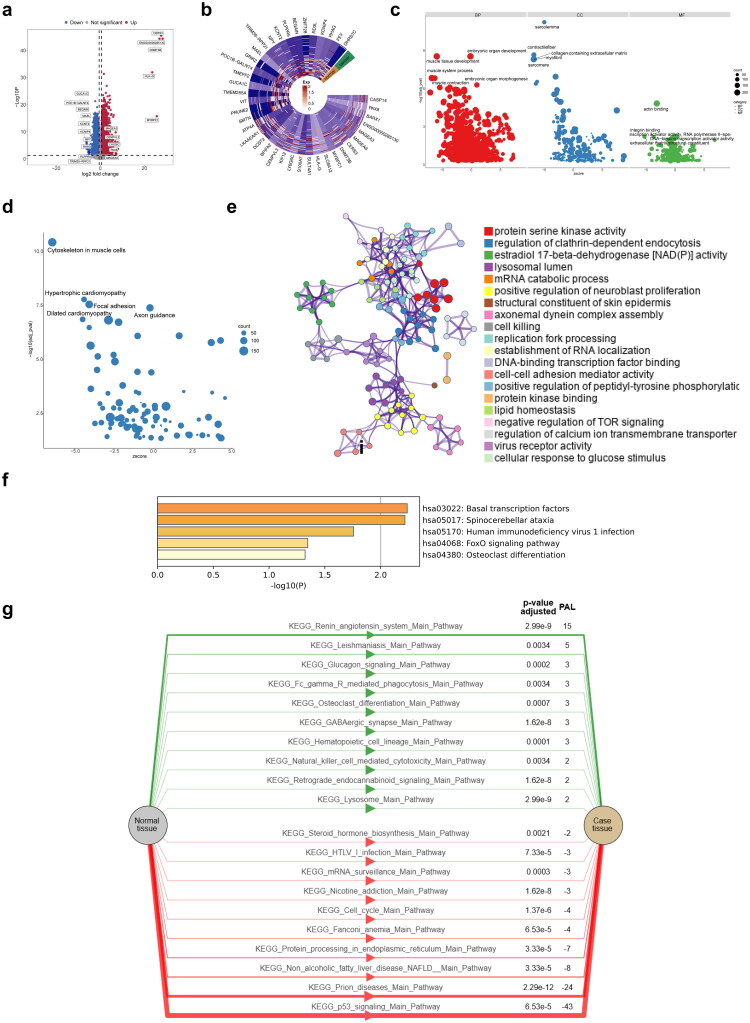
Identification and functional enrichment of DEGs and SMGs. **a** Volcano map of 7302 DEGs. **b** Heat map of the top 20 up-downregulated DEGs. **c** GO enrichment analysis showing the CC, BP, and MF involved in DEGs. **d** KEGG enrichment analysis showing the signaling pathways involved in DEGs. **e** GO enrichment analysis showing the CC, BP, and MF involved in SMGs. **f** KEGG enrichment analysis showing the signaling pathways involved in SMGs. **g** Levels of KEGG pathway activation in SMGs analyzed by OncoboxPD.

Differential expression analysis identified 7,302 DEGs between cancer and normal groups (Figure 1a,b; Table S2). GO enrichment analysis revealed significant terms related to sarcolemma, muscle tissue development, embryonic organ development, contractile fibers, and collagen-containing extracellular matrix (ECM) (Figure 1c; Table S3). KEGG enrichment analysis highlighted pathways such as the cytoskeleton in muscle cells, hypertrophic cardiomyopathy, focal adhesion, axon guidance, and dilated cardiomyopathy (Figure 1d; Table S4). WES of 35 BLCA tumor samples identified 78 SMGs, with missense mutations being predominant (Table S5). GO and KEGG enrichment analyses of SMGs revealed 139 GO terms and five KEGG pathways, including basal transcription factors, spinocerebellar ataxia, human immunodeficiency virus 1 infection, FoxO signaling pathway, and osteoclast differentiation (Figure 1e–f). KEGG enrichment using OncoboxPD showed activated pathways such as leishmaniasis (PAL = 5, *p* = 0.0034) and inhibited pathways such as p53 signaling (PAL = −43, *p* < 0.0001) (Figure 1g). These findings indicate that DEGs and SMGs were co-enriched in the FoxO signaling pathway in KEGG, suggesting that this pathway may play a central role in the regulation of both DEGs and SMGs in BLCA.

### 3.3. Identification and function of BLCA-associated DEMs and differential microbes

**Figure 2.**
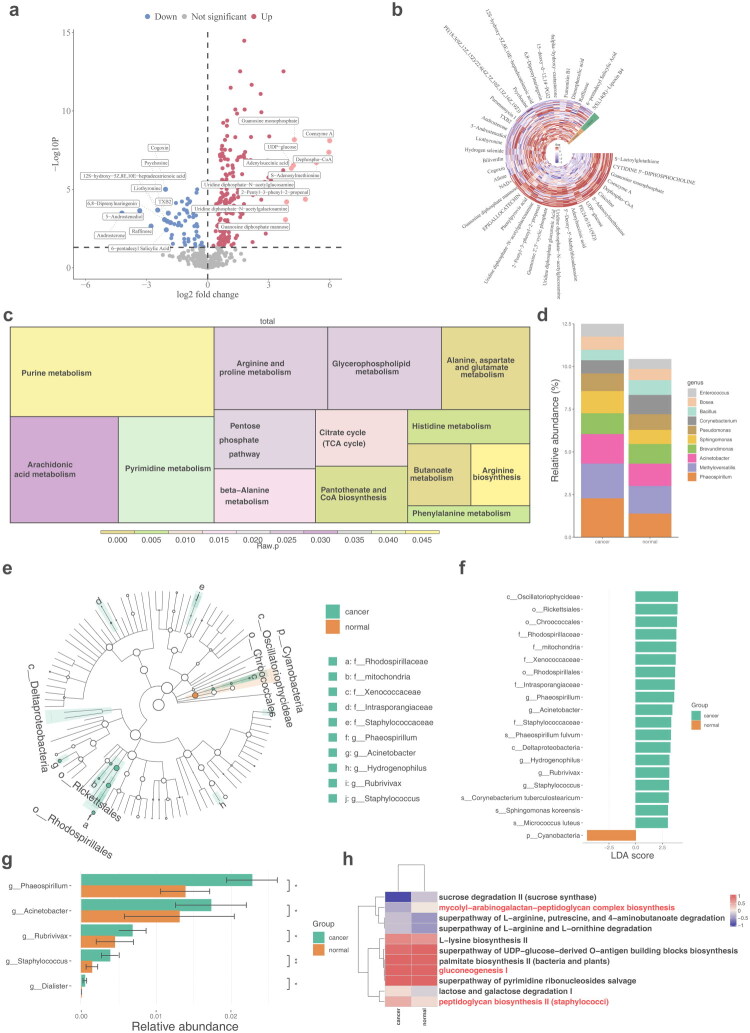
Identification of differential metabolites and differential microbiomes. **a** Volcano map of DEMs. **b** Heat map of DEMs. **c** KEGG enrichment analysis showing the signaling pathways involved in DEMs. **d** Intratumoural microbiome abundance at the genus level. **e** Branching plots showing abundance of differential microorganisms. **f** LDA scores for differential microorganisms. **g** Five differential microbiomes at the genus level. **h** KEGG enrichment analysis showing the signaling pathways involved in differential microbiomes.

Metabolome sequencing identified 212 DEMs, which were enriched in 14 KEGG pathways related to various amino acid metabolic processes (Figure 2a–c). To explore the distinctions in species diversity and composition of intratumoral microorganisms, both alpha and beta diversity analyses were performed across different sample groups. Alpha diversity, assessed using the Simpson, Shannon, InvSimpson, and Pielou, ACE and Chao1 indices, showed no significant differences between cancer and normal samples, indicating species diversity and evenness of distribution in both groups (Figure S3a). In the case of beta diversity, PCoA based on Bray–Curtis distance and UniFrac distance matrices showed that the microbial community structures of BLCA cancer tissues and adjacent tissues were separated, suggesting obvious differences in microbial composition between the two groups. However, significant separation was not observed in the analysis of Jaccard distance and Weighted UniFrac distance. In addition, the stress value was 0.11, indicating the reliability of the results (Figure S3b–c). The beta-diversity of the microbial communities between the two groups varied at the species abundance level, which might be associated with the niche changes of dominant bacterial genera in the tumor microenvironment. Intratumoral microbiome composition was examined at the genus levels. At the genus level, *Phaeospirillum* and *Methyloversatilis* were the most abundant (Figure 2d; Table S6). Using LEfSe analysis, five genera were identified that showed significant differences in abundance between cancer and normal samples: *Phaeospirillum*, *Acinetobacter*, *Rubrivivax*, *Staphylococcus*, and *Dialister* (Figure 2e–g). KEGG enrichment analysis of these microbiomes revealed significant pathways, including mycolylarabinogalactan–peptidoglycan complex biosynthesis, gluconeogenesis I, and peptidoglycan biosynthesis II (staphylococci) (Figure 2h).

### 3.4. Recognition of biomarkers

**Figure 3.**
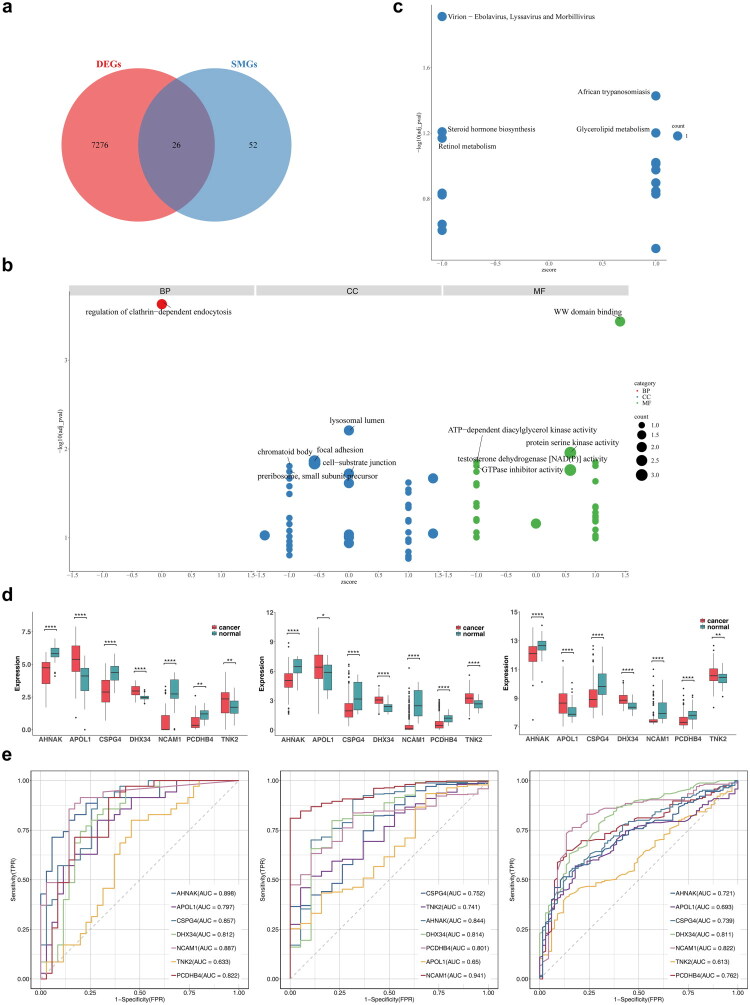
Identification and function analysis of candidate genes. **a** Identification of candidate genes. **b** GO enrichment analysis showing the CC, BP, and MF involved in candidate genes. **c** KEGG enrichment analysis showing the signaling pathways involved in candidate genes. **d** Expression analysis of seven differentially expressed candidate genes with prognostic value between groups in transcriptome sequencing data, TCGA–BLCA, and GSE13507 (Wilcoxon rank-sum test). * represented *p* < 0.05, ** represented *p* < 0.01, **** represented *p* < 0.0001. **e** ROC curve for the seven genes screened for five biomarkers with AUC > 0.7.

Cross-referencing DEGs and SMGs identified 26 candidate genes (Figure 3a), which were associated with processes such as clathrin-dependent endocytosis, WW domain binding, lysosomal lumen, protein serine kinase activity, and focal adhesion (Figure 3b). KEGG enrichment analysis highlighted significant enrichment in pathways related to Virion–Ebolavirus, Lyssavirus, Morbillivirus, and African trypanosomiasis (Figure 3c). Prognostic evaluation of these genes categorized TCGA–BLCA patients into high- and low-expression groups, with KM survival curves showing significant survival differences for 15 genes (Figure S4). Expression validation in the transcriptome, TCGA–BLCA, and GSE13507 datasets confirmed the up-regulation of APOL1, DHX34, and TNK2, and the down-regulation of *AHNAK*, *CSPG4*, *NCAM1*, and PCDHB4 in the cancer group (Figure 3d). ROC analysis identified *AHNAK*, *CSPG4*, DHX34, *NCAM1*, and PCDHB4 as biomarkers with AUC values greater than 0.7 across all datasets (Figure 3e).

### 3.5. Identification of key genes, metabolites, and microbiomes

**Figure 4.**
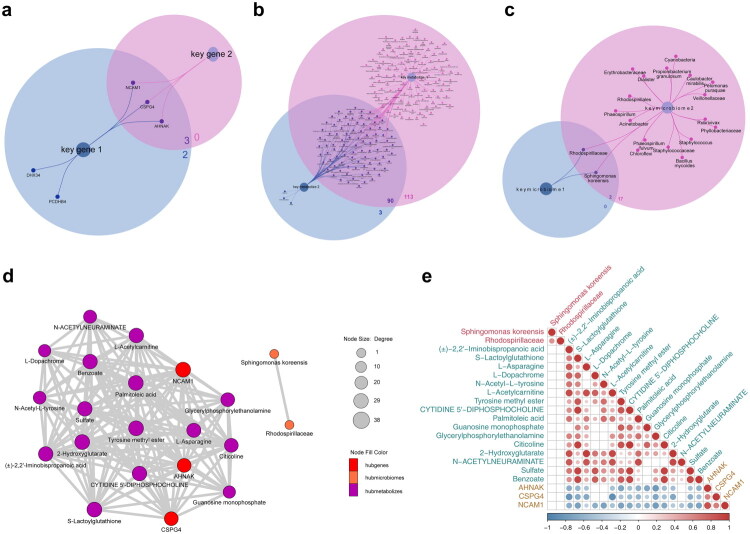
Identification of key genes, metabolites and microbiomes. **a** Three key genes from key gene 1 and key gene 2. **b** A total of 90 key metabolites from key metabolite 1 and key metabolite 2. **c** Two key microbiomes from key microbiome 1 and key microbiome 2. **d** The network of correlations between key genes metabolites microbiota. Red represented genes, orange represented microorganisms, and purple represented metabolites. **e** Heatmap showing relevance of key genes, metabolites, and key microbiomes. From blue to red, the correlation changed from negative to positive.

A combined analysis of biomarkers, DEMs, and differential microbiomes identified three key genes (*AHNAK*, *CSPG4*, *NCAM1*), 90 key metabolites, and two key microbiomes (Sphingomonas koreensis and Rhodospirillaceae) from multi-omics data (Figure S5; Figure 4a–c). Correlation and network analysis revealed positive correlations among the key genes, no correlation between key microbiomes and key genes or metabolites, and negative correlations between key genes and key metabolites (Figure 4d–e).

### 3.6. Multi-omic analysis reveals two blca subtypes linked to ecm and metabolism

**Figure 5.**
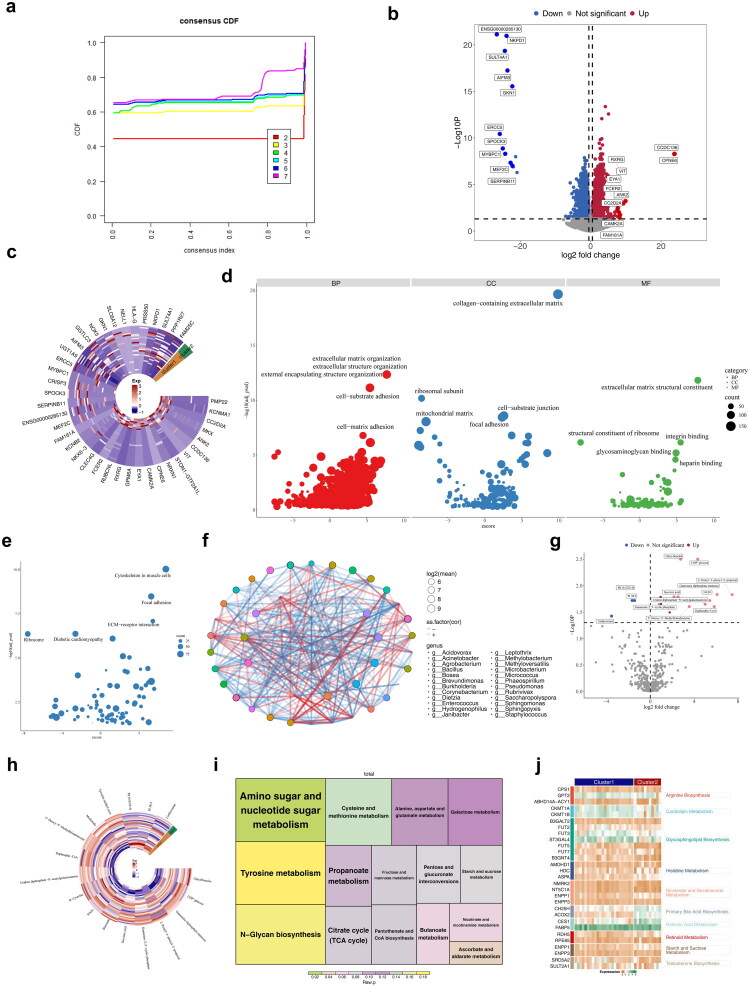
Analysis of differences among different clusters. **a** The cumulative distribution function (CDF) curve of consensus clustering. The *x* axis represents the consensus index, which reflects the clustering consistency between samples, while the y-axis represents the CDF value, which reflects the stability of the clustering results. Different colored curves corresponded to different numbers of clusters. **b** Volcano map of inter-cluster DEGs. **c** Heat map of the top 20 upregulated and downregulated DEGs between clusters. **d** GO enrichment analysis showing the CC, BP, and MF involved in inter-cluster DEGs. **e** KEGG enrichment analysis showing the signaling pathways involved in inter-cluster DEGs. **f** Association networks between microbial communities in different clusters. **g** Volcano map of inter-cluster differential metabolites. **h** Heat map of inter-cluster differential metabolites. **i** KEGG enrichment analysis showing the signaling pathways involved in inter-cluster differential metabolites. **j** GSVA results for two clusters. From green to brown, the expression of genes in each pathway was represented, with greener colors indicating higher expression.

BLCA samples were categorized into two clusters based on key gene expression (Figure 5a). A total of 3,464 DEGs were identified between clusters, which were enriched in 1,665 GO terms, with prominent categories including collagen-containing ECM and ECM organization (Figure 5b–d). A prognostic model of BLCA suggested that PD-L1 expression could predict patient prognosis, potentially linked to the ECM’s collagen passage [26]. KEGG enrichment analysis revealed 81 pathways, notably involving the cytoskeleton in muscle cells and focal adhesion (Figure 5e). Microbial community analysis indicated that most microbiomes within clusters were positively correlated (Figure 5f). Additionally, 17 differential metabolites between clusters were identified, enriched in amino sugar and nucleotide sugar metabolism, as well as ascorbate and aldarate metabolism (Figure 5g–i). GSEA revealed significant pathways across clusters, including the interferon-gamma response and epithelial–mesenchymal transition (EMT) (Figure S6). GSVA further identified 10 differential metabolic pathways (Figure 5j).

### 3.7. Tumor immune microenvironment (TIME) in BLCA clusters

**Figure 6.**
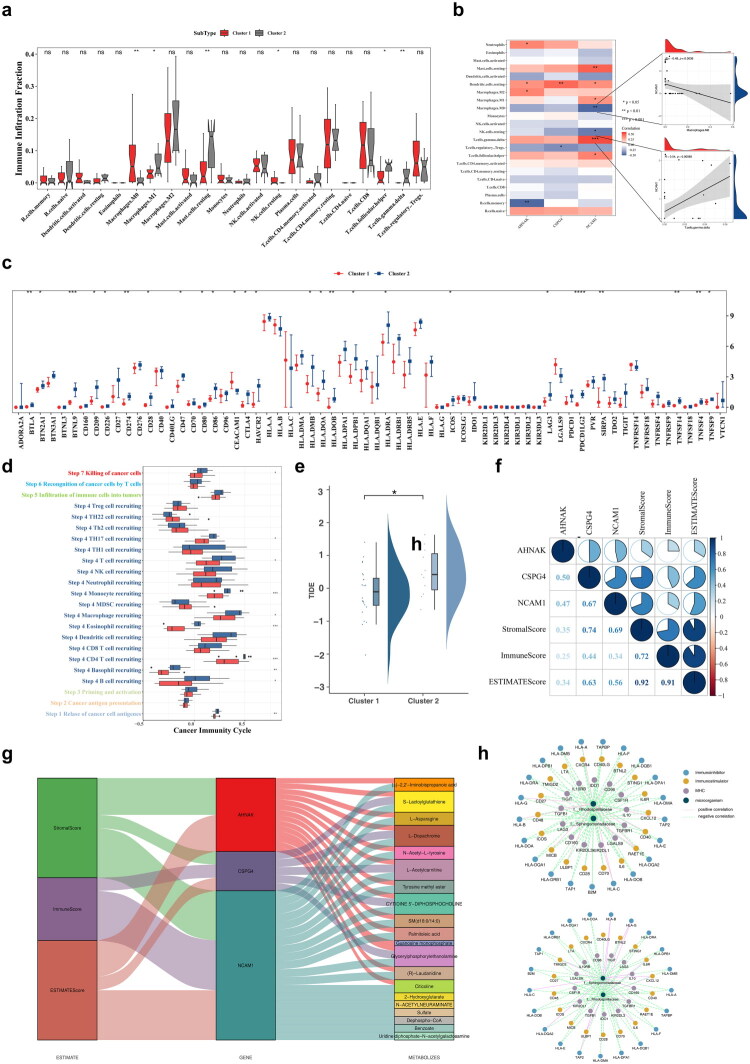
Tumour immune microenvironment in different clusters. **a** Differences in immune cell infiltration between cluster 1 and cluster 2 (Wilcoxon’s rank-sum test). ns represented no significance, * represented *p* < 0.05, ** represented *p* < 0.01. **b** Correlation of key genes with immune cells. * represented *p* < 0.05, ** represented *p* < 0.01, *** represented *p* < 0.001. **c** Differences in the expression of immune checkpoint genes between samples of different clusters (Wilcoxon rank-sum test). * represented *p* < 0.05, ** represented *p* < 0.01, *** represented *p* < 0.001, **** represented *p* < 0.0001. **d** Differences in the activity of circulatory pathways of cancer immunity between clusters (Wilcoxon rank-sum test). * represented *p* < 0.05, ** represented *p* < 0.01, *** represented *p* < 0.001. **e** TIDE score of cluster 1 and cluster 2 (Wilcoxon rank-sum test). * represented *p* < 0.05. **f** Correlations showing the relationship of key genes to stromal score, immune score and ESTIMATE score. **g** Association of immunotherapy, key genes and key metabolites. **h** Correlation network of key microorganisms with MHC molecules, immunostimulators, and immunoinhibitors. The upper image represented cluster 1, and the lower image represented cluster 2. Red lines indicated positive correlations, while green lines indicated negative correlations. Blue nodes represented immunosuppressants, brown nodes represented immune activators, purple nodes represented MHC molecules, and dark green nodes represented key microorganisms.

In the BLCA clusters, differential immune cell populations included M0/M1 macrophages, resting mast cells, resting natural killer cells, follicular helper T cells, and gamma delta T cells (Figure 6a). *NCAM1* was positively correlated with gamma delta T cells (Cor = 0.54) and negatively with M0 macrophages (Cor = −0.48) (Figure 6b). Among the 66 immune checkpoint genes analyzed, 25 showed differential expression between clusters, including BTLA, CD274, and CTLA4 (Figure 6c). Differences were observed in cancer immune cycle steps 1, 4, and 7 (Figure 6d). TIDE scores exhibited substantial divergence, reflecting distinct immune profiles between clusters (Figure 6e). Key genes were positively correlated with stromal, immune, and ESTIMATE scores, suggesting their relevance to immunotherapy (Figure 6f). These scores may also interact with key metabolites (Figure 6g). Key microbes enriched with immune factors showed negative correlations between BLCA clusters (Figure 6h).

### 3.8. BLCA subgroups exhibit distinct mutational profiles and therapy sensitivity

**Figure 7.**
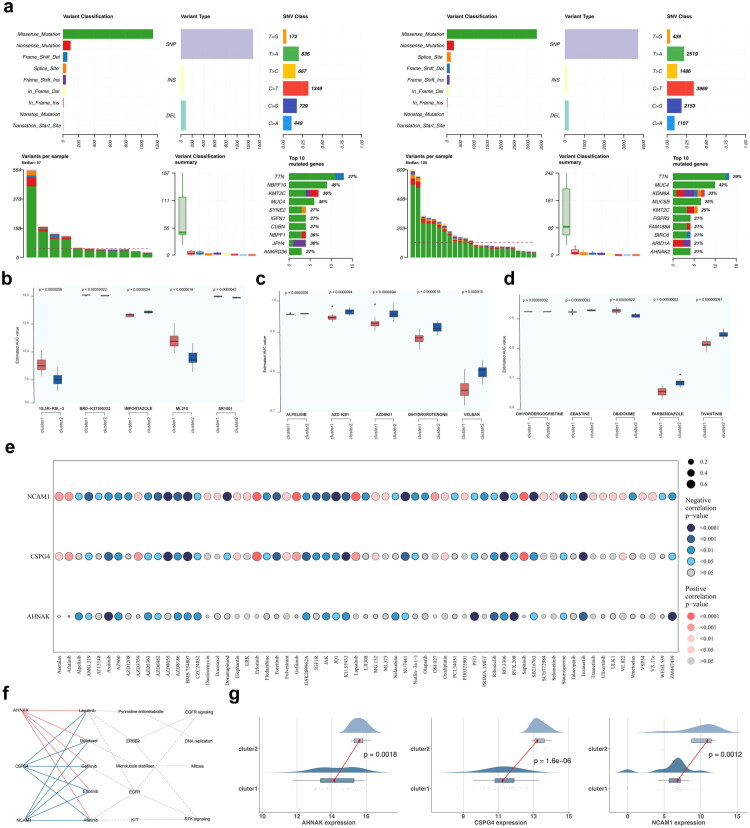
Gene mutations in clusters of BLCA. **a** Variant classification, variant type, and base mutations among different clusters. **b–c** Analysis of differences in drug response (AUC values) between clusters in CTRP (**b**), GDSC (**c**) and PRISM (**d**) databases (Wilcoxon rank-sum test). **e** Correlation between key genes and 63 chemotherapeutic agents. The color represented significance, with gray indicating no significance, red indicating a significant positive correlation, and blue indicating a significant negative correlation. The deeper the color, the more significant it was; the size of the points represented the strength of the correlation. **f** Correlation analysis among key genes, targeted agents, classical therapeutic pathways, and pathways. **g** Differences in the expression of three key genes between clusters.

The genomic mutation landscape of the top 20 most commonly mutated genes in BLCA provided detailed insights into CNV amplifications, deletions, mutation types, and base mutations. MUC4 exhibited the highest mutation frequency, with a significant proportion of missense mutations and C-to-T base substitutions (Figure 7a). Chemotherapy response analysis identified 236 differential agents in the CTRP database, 43 in GDSC, and 785 in PRISM Repurposing (Figure 7b–d). By comparing the 50% inhibitory concentration (IC50) values of common chemotherapeutic agents, such as FGFR and EGFR inhibitors, across different clusters, a total of 63 agents were found to differ significantly between clusters, most of which were strongly correlated with *NCAM1* expression (Figure 7e). Furthermore, key genes influenced classical therapeutic pathways as well as pathways targeted by corresponding agents (Figure 7f). Expression analysis revealed that key genes, including *AHNAK*, *CSPG4*, and *NCAM1*, exhibited differential expression between clusters, while 40 metabolites were differentially expressed, with no significant microbial differences observed (Figure 7g; Figure S7).

### 3.9. Exploration of key gene-related mechanisms

**Figure 8.**
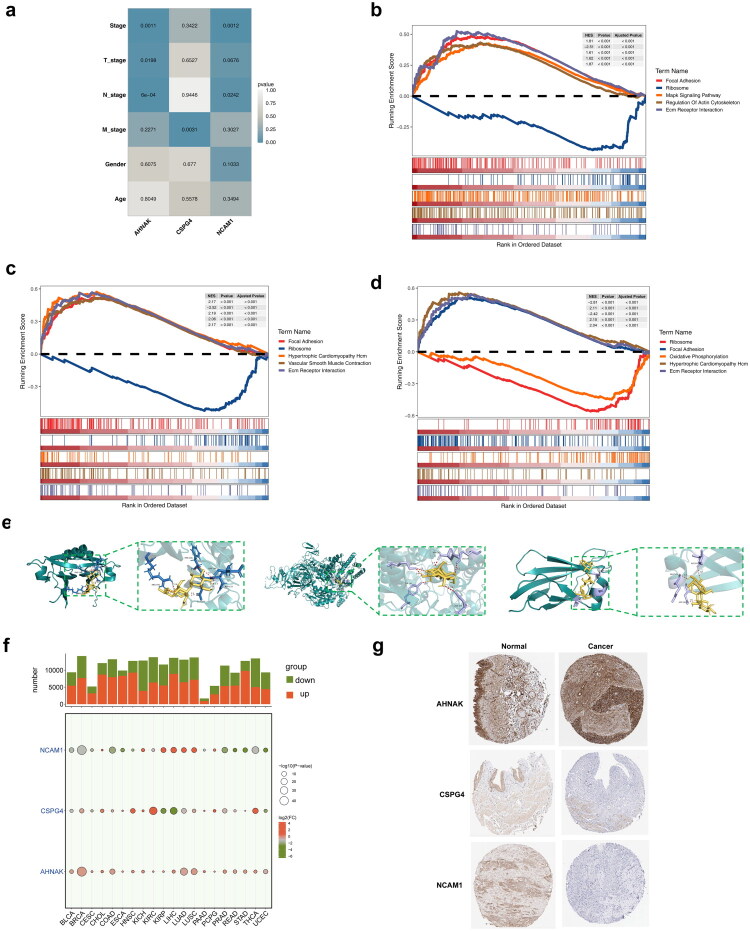
Expression of key genes. **a** Correlation of key genes with clinical characteristics. **b–d** KEGG enrichment analysis showing the signaling pathways involved in three key genes. Three key genes were *AHNAK* (**b**), *CSPG4* (**c**), and *NCAM1* (**d**). **e** Molecular docking of key genes (*AHNAK*, *CSPG4*, and *NCAM1*) and maltotriose. **f** Expression of individual key genes in pan-cancer data. **g** Expression of key genes (*AHNAK*, *CSPG4*, and *NCAM1*) at the protein level in cancer and normal samples.

To analyze key gene expression in BLCA, patients were categorized based on clinical features from TCGA–BLCA. Both *AHNAK* and *NCAM1* displayed differential expression in relation to T stage, N stage, and overall stage, while *CSPG4* showed limited differential expression only in relation to stage (Figure S8). *AHNAK* exhibited a positive correlation with N stage, T stage, and overall stage, while *CSPG4* negatively correlated with M stage. *NCAM1* showed positive correlations with both stage and N stage (Figure 8a; Figure S9). Survival analyses indicated significant differences in outcomes based on high and low expression levels of these key genes (Figures S10–S12). Single-gene GSEA identified significant pathways such as focal adhesion, ribosome function, and ECM receptor interactions (Figure 8b–d). *AHNAK*, *CSPG4*, and *NCAM1* were enriched in 35, 59, and 31 pathways, respectively, related to olfactory transduction, NKCC, and CAMs. Maltotriose and other compounds showed potential as therapeutic agents, with excellent docking interactions of −6.1 kcal/mol for *AHNAK*, −8.5 kcal/mol for *CSPG4*, and −6 kcal/mol for *NCAM1* (Figure 8e). Additionally, expression data revealed that the three key genes varied across most cancers (Figure 8f; Figure S13). Further analysis through NCBI and the HPA database revealed high expression of these genes in the skin, colon, and cerebral cortex. At the protein level, however, no significant differences in expression were observed between cancer and normal samples (Figure 8g).

### 3.10. Expression of key genes at the single-cell level

**Figure 9.**
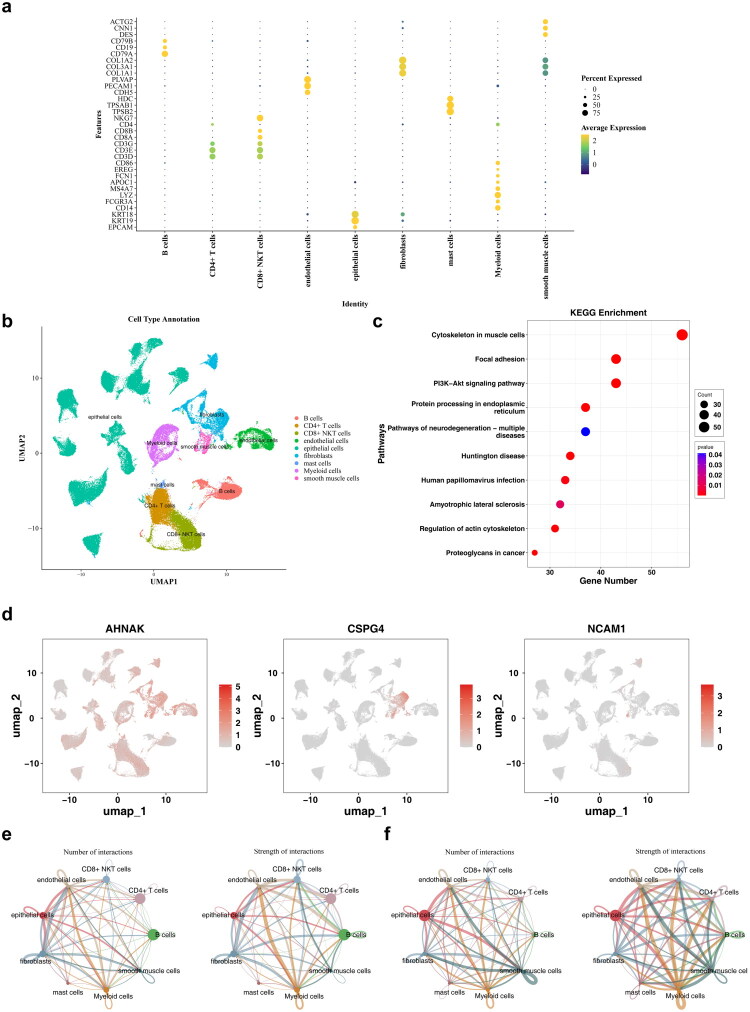
Single-cell analysis. **a** The bubble plot of cell-type-specific highly expressed genes. **b** The UMAP plot of cell type annotations. **c** The bubble plot of the top 10 KEGG enriched pathways. **d** The distribution of core genes across different cell types. **e** Cell communication results in normal samples. The left panel showed the intensity distribution of interactions between different cell types, and the right panel presented the statistical count of interactions among various cell types. **f** Cell communication results in tumor samples.

**Figure 10.**
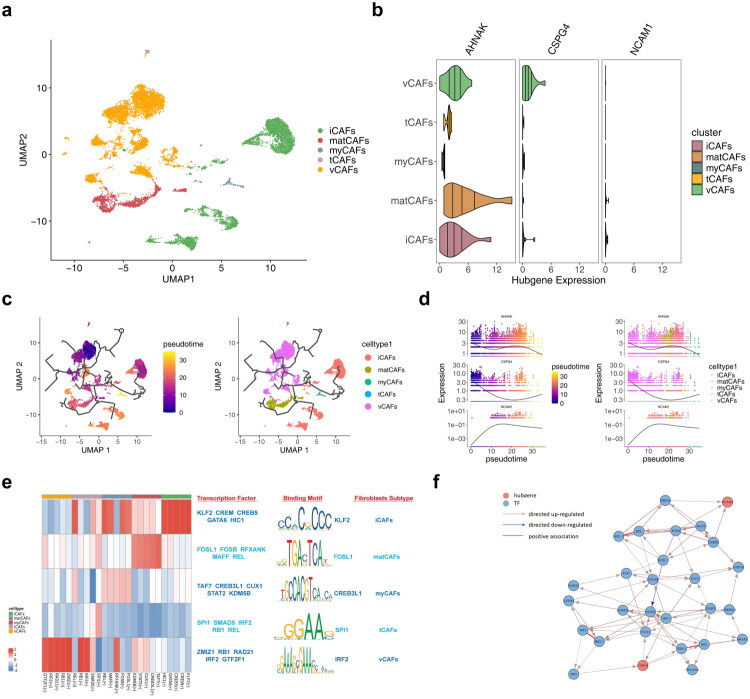
Pseudotime analysis in fibroblasts. **a** Annotation analysis of fibroblast subpopulations. **b** Expression analysis of core genes in various cell subpopulations. **c** Pseudotime analysis of key cells. **d** Expression of core genes in different pseudotime stages of key cells’ differentiation trajectory. **e** Core regulatory modules and representative transcription factors and binding motifs in each cell subpopulation of key cells. **f** Directed network plot between transcription factors and core genes. Red represented core genes, blue represented transcription factors, red lines indicated positive correlations, red arrows indicated positive regulation, and blue lines indicated negative regulation.

Following quality control, the single-cell sequencing dataset included 130,431 cells and 36,137 genes (Figure S14a,b). From this, 2,000 highly variable genes were selected, and 32 cell clusters were identified using UMAP (Figure S14c–e). Nine distinct cell types were annotated: CD4^+^ T cells, endothelial cells, fibroblasts, myeloid cells, smooth muscle cells, epithelial cells, B cells, CD8^+^ natural killer cells, and mast cells (Figure 9a,b) Key cell types were primarily involved in pathways such as the cytoskeleton in muscle cells, focal adhesion, PI3K–Akt signaling, and protein processing in the endoplasmic reticulum (Figure 9c). *AHNAK* was broadly expressed across cell types, while *CSPG4* was predominantly found in fibroblasts and smooth muscle cells, and *NCAM1* exhibited minimal expression (Figure 9d). Therefore, fibroblasts were designated as key cells for further analysis. Significant differences in intercellular communication were observed between normal and tumor cells (Figure 9e–f). Given these findings and supporting literature, fibroblasts were selected for further investigation and were reclustered into iCAFs, matCAFs, myCAFs, tCAFs, and vCAFs (Figure 10a). *AHNAK* expression was notably higher in fibroblast subtypes (Figure 10b). Pseudo-time trajectory analysis showed that *AHNAK* expression decreased, *CSPG4* varied, and *NCAM1* initially increased before decreasing (Figure 10c,d). Transcription factors and binding motifs associated with key cell subtypes were identified (Figure 10e). Additionally, the potential regulatory effects between transcription factors and key genes were inferred and illustrated through directed networks (Figure 10f).

**Figure 11.**
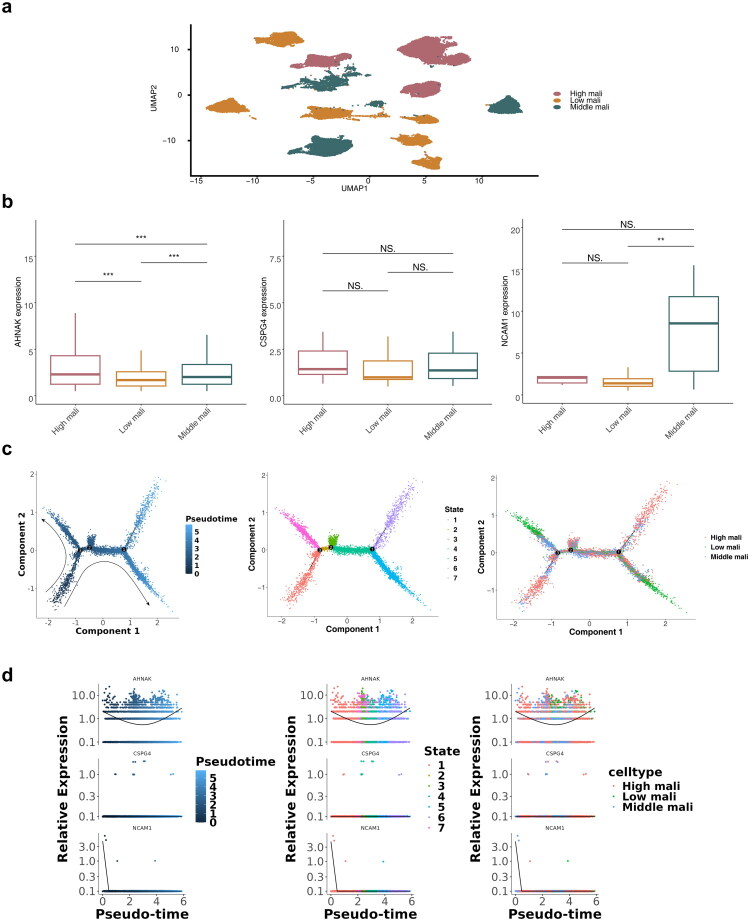
Analysis of cellular malignancy. **a** UMAP for distribution of high malignancy, middle malignancy and low malignancy. **b** Expression analysis of key genes in subclusters of cells with different degrees of malignancy. NS represented no significance, ** represented *p* < 0.01, *** represented *p* < 0.001. **c** Pseudo-time analysis of epithelial cells. **d** Expression of key genes during different pseudo-time and subcluster differentiation of cells with different degrees of malignancy.

BLCA primarily originates from epithelial cells. To explore malignancy further, epithelial cells were categorized into high, intermediate, and low malignancy groups based on CNV scores of cell subclusters (Figure 11a). Notably, *AHNAK* gene expression varied across cell subpopulations with differing malignancy levels, while *CSPG4* and *NCAM1* remained unchanged in the low and moderate malignancy groups (Figure 11b). Differentiation trajectory analysis revealed that *AHNAK* expression initially decreased and then increased over time, while *CSPG4* remained stable and *NCAM1* decreased to a point before stabilizing (Figure 11c,d).

## 4. Discussion

BLCA, one of the most prevalent malignancies of the urinary tract, primarily affects individuals over the age of 55, with a higher incidence in men [39]. As the understanding of BLCA pathogenesis has advanced, numerous studies have refined molecular classification based on gene and protein expression, while also developing corresponding prognostic models [40–42]. However, tumorigenesis remains a complex process driven by multiple biological pathways. To provide a more comprehensive and accurate molecular subtyping for patients with BLCA, WES data were combined with intratumoral microbiome data. Three key genes, 90 key metabolites, and two key microbiota were identified through DEGs, SMGs, and DEMs. A gene–metabolite–microbe regulatory network was then constructed. Additionally, BLCA samples were categorized into two subgroups based on key genes, which displayed significant differences in immunity, gene mutations, drug sensitivity, and clinicopathological features.

The three key genes selected—*AHNAK*, *CSPG4*, and *NCAM1*—were significantly underexpressed in patients with BLCA, with expression levels negatively correlating with OS. Despite their limited exploration in previous studies, these genes play vital roles in BLCA pathogenesis [43,44]. Ankyrin repeat domain-containing protein 1 (*AHNAK*), a cytoskeletal protein, is essential for calcium homeostasis, muscle formation, and various biological processes such as cell proliferation and signaling [45]. KEGG enrichment analysis highlighted the association of *AHNAK* with cell adhesion and the cytoskeleton in BLCA. *AHNAK* primarily acts as an inhibitory oncogene in BLCA, with significantly reduced expression in tumor individuals. Additionally, *AHNAK* levels in urine could serve as a biomarker to differentiate between uroepithelial carcinoma and normal uroepithelial cells [46], which aligns with our findings. Previous studies have demonstrated that NAT10 promotes cisplatin resistance in bladder cancer by indirectly facilitating DNA damage response (DDR) through post-transcriptional regulation of AHNAK mRNA stability [46]. Chondroitin sulfate proteoglycan 4(*CSPG4*), a transmembrane protein, is aberrantly expressed in various cancers and has been implicated in tumor invasion and lymphovascular infiltration [47]. In BLCA, *CSPG4* is underexpressed, influencing EMT, immune microenvironment, and energy metabolism, with a negative correlation to patient prognosis [48]. Our findings support these conclusions, suggesting *CSPG4*’s involvement in cell migration and invasion in BLCA, as well as its potential role in regulating key metabolites and microbiota. Neural Cell Adhesion Molecule 1 (*NCAM1*), a member of the immunoglobulin superfamily of adhesion molecules, is associated with neurogenesis, synapse growth, cell proliferation, and migration in various cancers such as gliomas, ovarian carcinomas, and small-cell lung carcinomas [49]. It has also been identified as a potential prognostic and therapeutic target in BLCA [43]. Our immunoinfiltration analysis revealed a significant positive correlation between *NCAM1* and γδ T cells, suggesting its potential role in modulating anti-tumor immunity. Specifically, *NCAM1* may leverage its adhesion properties to facilitate γδ T cell recruitment within the TME, thereby reshaping the local immune landscape and enhancing immune surveillance against tumor cells [50]. These findings collectively demonstrate *NCAM1*’s potentially critical immunoregulatory functions, warranting further mechanistic validation to establish the *NCAM1*-γδ T cell axis as a targetable pathway for immunotherapy development.

Through the construction of an integrated network incorporating key genes, metabolites, and microbial features, this study revealed a negative correlation between *AHNAK* and glycolysis- and lipid metabolism-related metabolites. Existing evidence indicates that *AHNAK* overexpression activates the PI3K–Akt signaling pathway [51], which serves as a central metabolic regulator and may suppress glycolysis *via* downstream effectors, thereby reducing glycolytic metabolite production [52] and impairing tumor cell survival and invasiveness. Notably, palmitoleic acid emerged as a critical node connecting glycolysis and lipid metabolism networks. Mechanistically, *AHNAK* may disrupt intracellular lipid homeostasis *via* PI3K–Akt signaling, compromising membrane stability/function and ultimately modulating tumor cell viability and metastatic potential. Consistent with previous studies [7], the analysis highlighted key metabolites enriched in amino acid and nucleotide metabolic pathways, such as purine, arginine, glycerophospholipid, and pyrimidine metabolism, based on KEGG results. High extracellular adenosine deaminase activity in BLCA cell lines, as demonstrated by Hesse et al. [53], interfered with antitumor immune responses by altering purine metabolism and impacting adenosine levels. Arginine metabolism also plays a significant role in antitumor immunity, primarily by influencing components of the tumor microenvironment, such as macrophages and T cells [54]. The pyrimidine metabolism-related enzyme UPP1 is essential for maintaining uridine homeostasis and has been shown to promote BLCA cell proliferation and gemcitabine resistance through the activation of the AKT signaling pathway [55]. Additionally, functional enrichment analysis of differential microbes demonstrated associations with arginine and pyrimidine metabolism pathways. The key microbes, Sphingomonas koreensis and Rhodospirillaceae, are environmental bacteria with potential roles in both health and disease. Sphingomonas koreensis was first identified as a meningitis pathogen in 2015 [56]. Reduced levels of Rhodospirillaceae correlate with high metastatic potential in pancreatic cancer, although the exact mechanisms underlying its role in cancer progression remain unclear, potentially involving interactions with the host immune system and bacterial metabolite production [57]. Furthermore, accumulating evidence highlights the pivotal role of microbiota–immune interactions in bladder cancer progression [58]. The microbiota modulates host immune responses, not only influencing oncogenesis but also synergizing with existing clinical therapies to improve the management of urothelial malignancies [59–61]. These findings underscore the multifaceted role of microbial communities in immune regulation and therapeutic response, suggesting that deeper mechanistic insights into tumor-associated microbiota may yield novel diagnostic and therapeutic strategies.

Patients with BLCA were classified into two clusters based on the expression of three key genes, and the functional differences between these clusters were analyzed. This analysis revealed that the pathways enriched in the subtypes primarily involved the ECM, cytoskeleton, adhesion mechanisms, and various inflammatory and tumor-related biological processes, consistent with the functional enrichment results of the key genes. The ECM, a critical component of the tumor microenvironment, exhibits altered collagen composition that is linked to EMT, facilitating cell adhesion, migration, and the progression of BLCA [62]. Additionally, a positive correlation between BLCA subtypes and a wide array of microbes was observed. Previous studies have documented the interaction between microbes and the ECM in the tumor microenvironment, where specific bacterial strains disrupt tight junctions within ECM components, enabling tissue colonization and inflammation. This promotes ECM remodeling and generates reactive oxygen species, which can induce DNA damage and contribute to cancer recurrence [63]. Therefore, the identified BLCA subtypes may influence tumor progression through microbial interactions with the ECM. GSEA highlighted clustering around common tumor-related pathways, including EMT, interferon-gamma response, TNFA signaling *via* NFkB, IL6 JAK STAT3 signaling, and the p53 pathway, all of which play pivotal roles in BLCA growth, progression, DNA repair, and response to immunotherapy [64–66]. Immune infiltration and mutation profiles were further examined across subtypes, revealing notable differences in immune microenvironments and oncogenic mutation patterns. Cluster 2 exhibited higher levels of immune infiltration and checkpoint expression, yet displayed reduced responsiveness to chemotherapeutic agents such as FGFR and EGFR inhibitors. Tumor cell growth and development are significantly influenced by interactions with the surrounding microenvironment; a high TMB results in the production of aberrant proteins that are recognizable by immune cells, triggering an effective anti-tumor immune response [67]. Simultaneously, immune cell infiltration modulates this response, directly correlating with tumor progression and therapeutic outcomes [68].

Through single-cell analysis, we identified cancer-associated fibroblasts (CAFs) as a key cellular component and further classified them into five distinct subtypes (iCAFs, matCAFs, myCAFs, tCAFs, and vCAFs), each demonstrating unique gene expression patterns and functional specialization. Notably, *AHNAK* showed significant overexpression in both iCAFs and matCAFs. The iCAF subtype contributes to the formation of an inflammatory microenvironment by regulating cell adhesion and tight junctions while secreting various cytokines, which aligns with previous findings by Chen et al. [69] showing that such microenvironment can reduce chemosensitivity in BLCA patients and enhance tumor invasion and metastatic potential. Meanwhile, the elevated *AHNAK* expression in matCAFs appears to cooperate with their ECM-synthesizing function, facilitating tumor growth through secretion of collagen and other components to establish a tumor-supportive stromal structure [70]. Pathway enrichment analysis further confirmed that CAFs mediate tumor proliferation and drug resistance by driving EMT, ECM remodeling, angiogenesis, and suppression of anti-tumor immunity [71]. Additionally, epithelial cells, as the dominant cell type in BLCA tissues, can adopt a malignant phenotype under certain conditions [72]. A subset of epithelial cells expressing N-calmodulin 2 (CDH12) demonstrated specific invasive traits, such as chemoresistance and poor prognosis [73]. Consequently, the degree of malignancy in epithelial cells was evaluated, revealing that *AHNAK* expression was negatively correlated with malignancy. This is consistent with *AHNAK*’s role as a cytoskeletal protein, which may influence cellular dynamics in BLCA. These findings significantly advance our understanding of CAF heterogeneity by elucidating the functional divergence among distinct CAF subtypes in BLCA, while providing novel insights for developing precision therapies targeting specific CAF subpopulations and their key molecular determinants.

In summary, this study provides novel insights into the biological mechanisms of BLCA through an integrative multi-omics approach incorporating transcriptomics, WES, metabolomics, intratumoral microbiome profiling, and single-cell data. Furthermore, our work significantly advances the understanding of CAF heterogeneity by systematically characterizing, for the first time, the distinct gene expression patterns and functional specialization of CAF subtypes, particularly their subtype-specific molecular signatures in BLCA pathogenesis. The observed negative correlations between key genes and metabolites suggest these genes may drive BLCA progression through metabolic pathway regulation. These findings not only elucidate the critical roles of CAF subtypes in BLCA biology but also identify potential therapeutic targets for future precision medicine strategies. However, several limitations of the study remain. First, The relatively small sample size may compromise the statistical power of the results, and the lack of MIBC subtype classification in the patient cohort limits a comprehensive evaluation of the impact of subtype differences on tumor aggressiveness, microenvironment complexity, and prognosis. Furthermore, international guidelines explicitly recommend considering variant histologic types [74,75], yet this study did not incorporate bladder cancer subtype classification, potentially introducing bias in the analysis of key genes associated with tumor invasiveness. Additionally, the datasets (e.g. GSE13507) lacked critical clinical information such as age, and incomplete clinical characteristics of the samples may affect the reliability of the findings. Multiple testing correction was not uniformly applied, which could influence the statistical outcomes. Moreover, LEfSe may have inherent limitations when handling compositional data, and the selected marker genes in the single-cell analysis might not fully capture tumor heterogeneity. Therefore, future studies should aim to collect larger sample sizes, incorporate multicenter clinical data and single-cell data encompassing diverse subtypes, and perform more comprehensive analyses of the associations among genes, metabolites, microbiota, and clinical features. Mixed-effects models could be employed to assess interpatient cellular correlations, supplemented by functional experiments for validation. Adopting stricter statistical methods (e.g. multiple testing correction) and more appropriate analytical approaches for compositional data (e.g. ANCOM, ANCOM-BC, and ALDEx2) would enhance the robustness of the research. Additionally, future investigations should align with the 2022 WHO classification criteria and leverage multi-omics integrative analyses to further elucidate the molecular characteristics of different subtypes [76,77], exploring their prognostic and therapeutic implications to advance precision medicine. By constructing Cox proportional hazards models incorporating key genes, metabolites, and microbial interactions, this approach may provide novel insights for the early diagnosis and personalized treatment of bladder cancer.

## 5. Conclusion

This study utilized an integrated multi-omics approach to identify significant correlations between three pivotal genes—*AHNAK*, *CSPG4*, and *NCAM1*—and key metabolites and microorganisms in BLCA. These findings offer novel insights into the molecular mechanisms of BLCA and provide a comprehensive framework for developing targeted therapeutic strategies. The identified biomarkers and their interactions could improve diagnostic accuracy, enable personalized treatments, and further our understanding of BLCA, ultimately improving patient outcomes.

## Supplementary Material

Supplemental Material

Supplementary Tables.xlsx

## Data Availability

Derived data supporting the methodology of this study are available from the corresponding author upon reasonable request.
