# Supplementary material for: Construction of a gene–metabolite–microbiome regulatory network reveals novel therapeutic targets in bladder cancer through multi-omics analysis
Source: Ann Med. 2025 Sep 5;57(1):2553220. doi: 10.1080/07853890.2025.2553220 (PMC12416030; doi:10.1080/07853890.2025.2553220)
Supplement: Supplemental Material [file IANN_A_2553220_SM7070.docx]

**Supplementary Figures:**

**
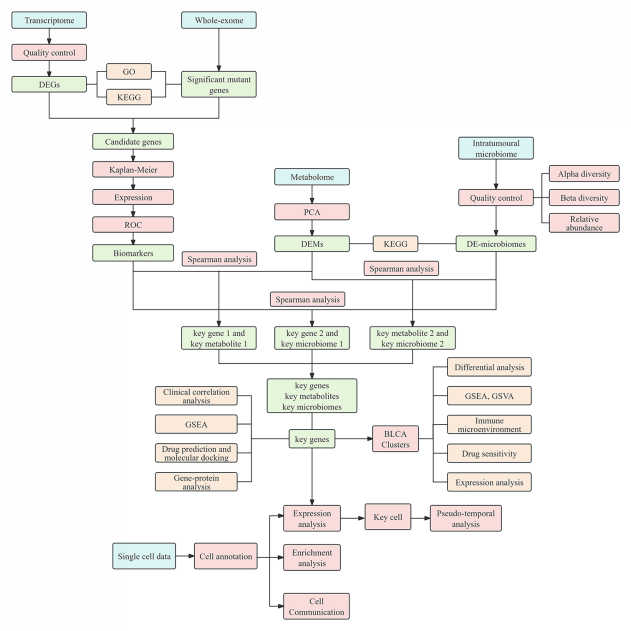
**

**Fig.S1: The flowchart of the analysis in this study.**

**
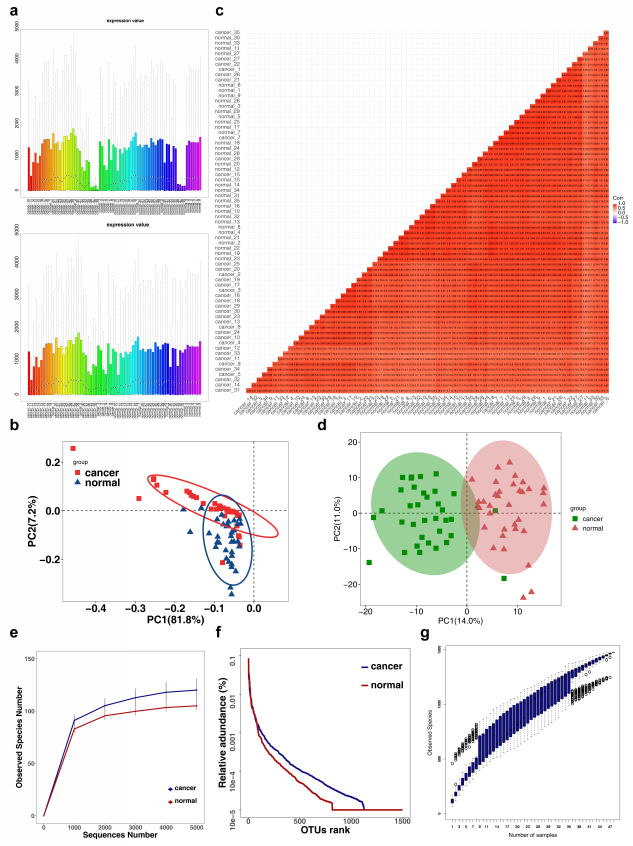
**

**Fig.S2: Analysis of sequencing data.**

**a** Expression of transcriptome samples. The cancer and paracancerous tissue samples from four BLCA patients, numbered 36, 37, 38 and 39 were excluded, the upper figure is before exclusion the lower figure is after exclusion. **b** PCA for two groups of samples from transcriptome sequencing data. **c** Correlation analysis between samples. **d** PCA for two groups of samples from metabolome sequencing data. **e** Rarefaction curves for intratumoural microbiome sequencing data. **f** The microbial community rank abundance curves of tumor and adjacent normal samples. The x-axis represented the OTUs ranks arranged in descending order of relative abundance, while the y-axis represented the relative abundance of microorganisms (%). The slope of the curve reflected species abundance evenness. The slope of the tumor sample curve was steeper, indicating a higher proportion of dominant species and lower evenness in the microbial community. In contrast, the slope of the normal sample curve was more gradual, suggesting a more balanced distribution of species abundance. **g** The species cumulative box plot.

**
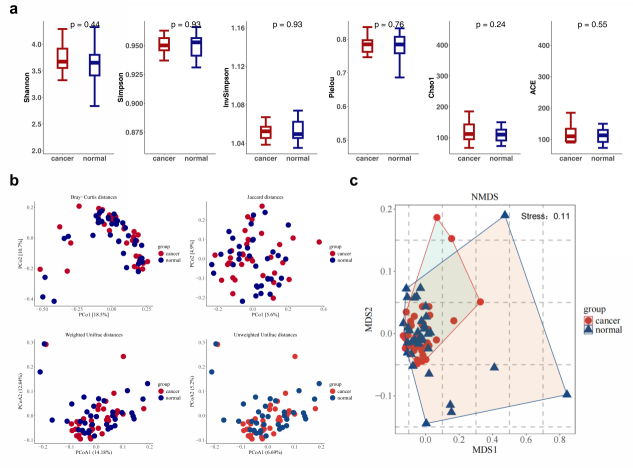
**

**Fig.S3: Microbiological diversity analysis.**

**a** Alpha diversity for intratumoural microbial with the Simpson, Shannon, InvSimpon, Pielou, ACE and Chao1 indices. **b** Principal coordinate analysis of Beta diversity in intratumoral microorganisms by Bray-Curtis, Jaccard, Weighted Unifrac, and Unweighted Unifrac distances. **c** Non-metric MultiDimensional Scaling analysis between the two sample groups.


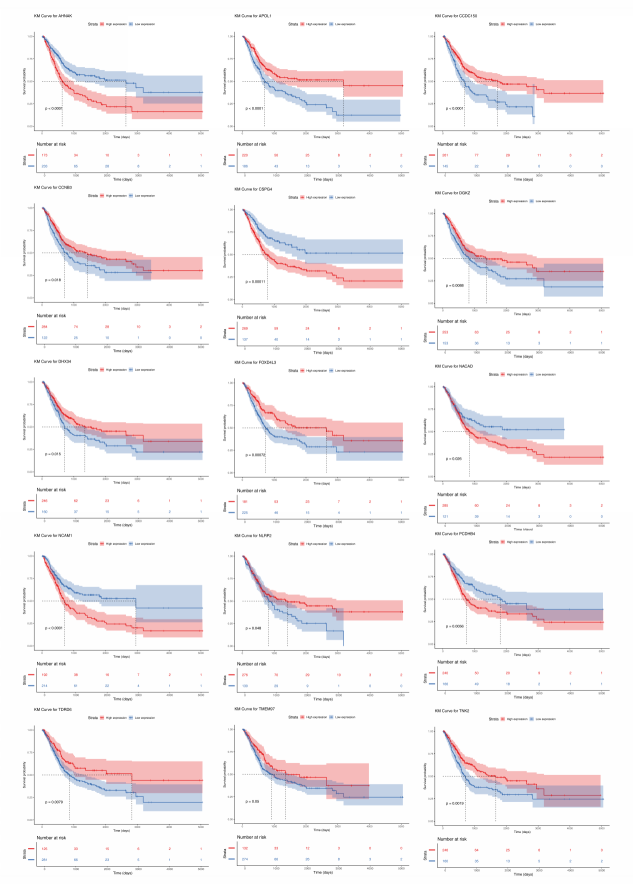


**Fig.S4: Kaplan-Meier survival curves showing significant survival differences for 15 genes.** The genes were arranged from left to right as follows: *AHNAK*, *APOL1*, *CCDC150*, *CCNB3*, *CSPG4*, *DGKZ*, *DHX34*, *FOXD4L3*, *NACAD*, *NCAM1*, *NLRP2*, *PCDHB4*, *TDRD6*, *TMEM97*, and *TNK2*.

**
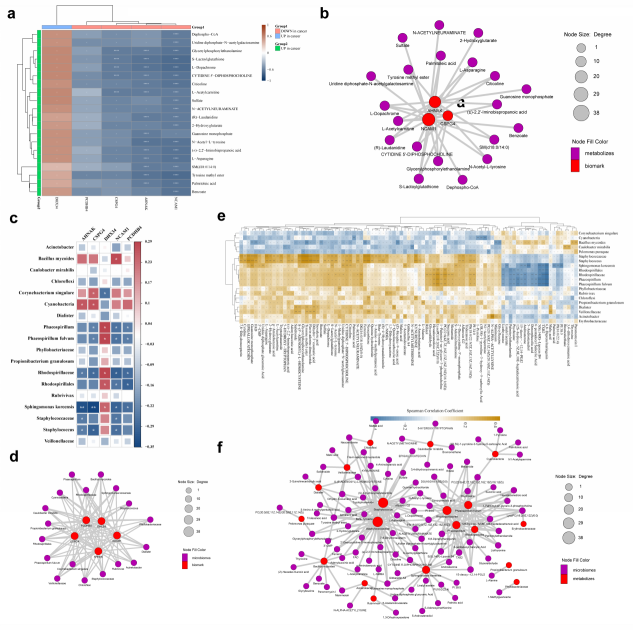
**

**Fig.S5: Correlation analysis.**

**a, b** Spearman analysis between biomarkers and DEMs for key gene 1 and key metabolite 1. **c, d** Spearman analysis between biomarkers and differential microbes for key gene 2 and key microbiome 1. **e, f** Spearman analysis between DEM and differential microbes for key metabolite 2 and key microbiome 2.


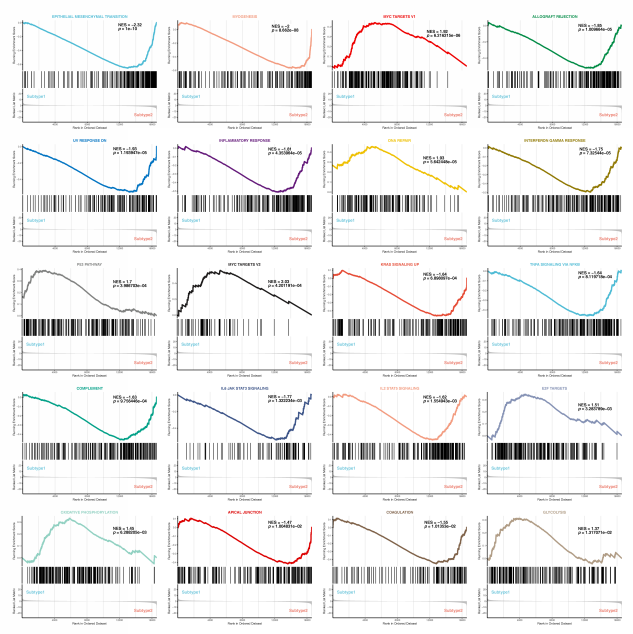


**Fig.S6: GSEA results with top 20 for two clusters.** The top part was the ES score curve, with the score at the highest/lowest points representing the ES value of the pathway. If the ES value of a pathway was positive, it indicated activation in subtype 1. The middle part displayed the position of genes in the pathway within the ranking list, with each vertical line representing a gene. The bottom part was the logFC distribution plot, with the x-axis representing genes and the y-axis representing logFC.


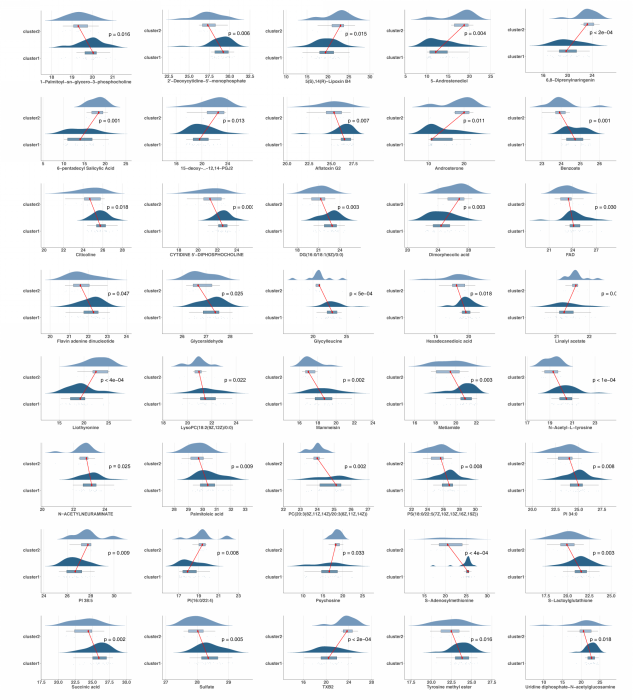


**Fig.S7: Differences in expression of key metabolites between BLCA clusters.**

**
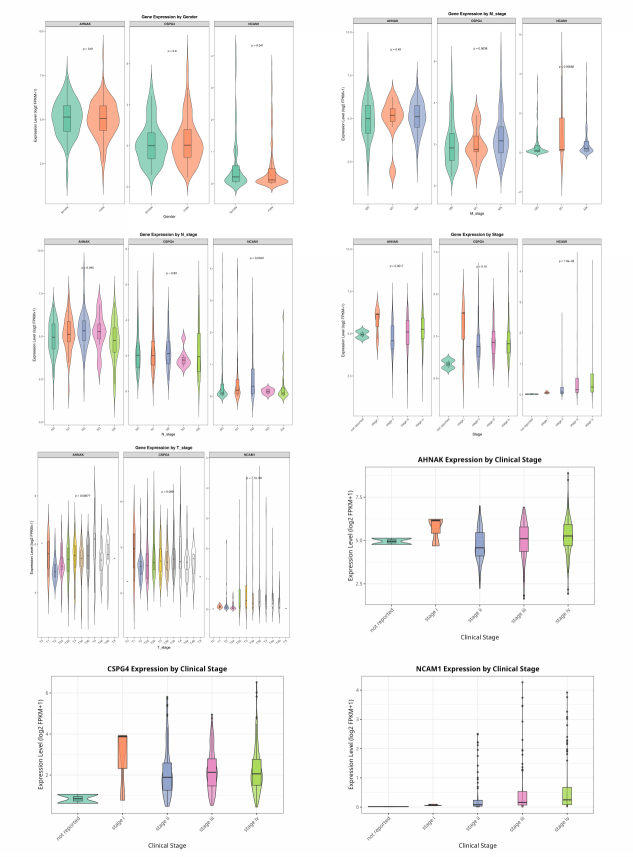
**

**Fig.S8: Association analysis of clinical characteristics of key genes.**

**a** Analysis of *AHNAK* expression between different clinical subgroups in the TCGA-BLCA dataset. **b** Analysis of *CSPG4* expression between different clinical subgroups in the TCGA-BLCA dataset. **c** Analysis of *NCAM1* expression between different clinical subgroups in the TCGA-BLCA dataset.

**
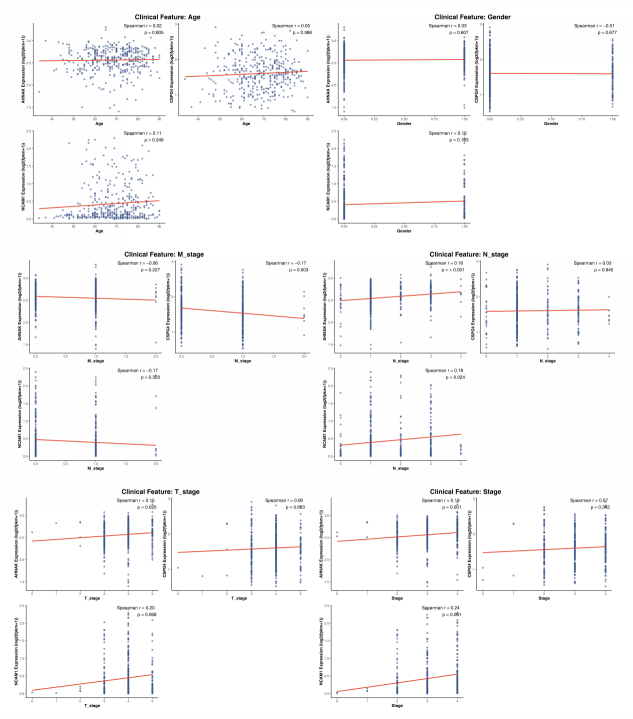
**

**Fig.S9: The scatter plot of the correlation between core genes and clinical features.**

**
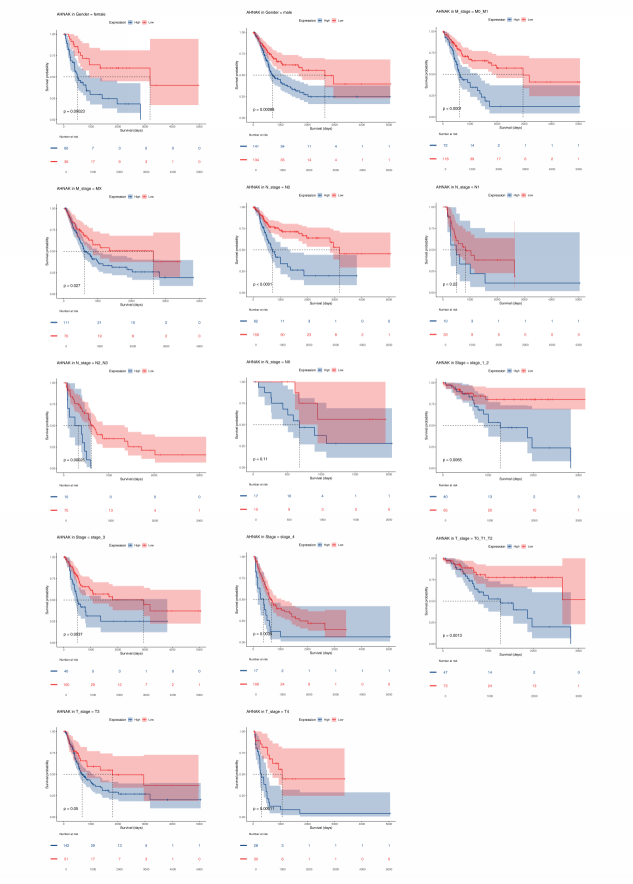
**

**Fig.S10: KM survival curves showing overall survival of patients in high and low expression sets of *AHNAK* in different clinical subgroups.**

**
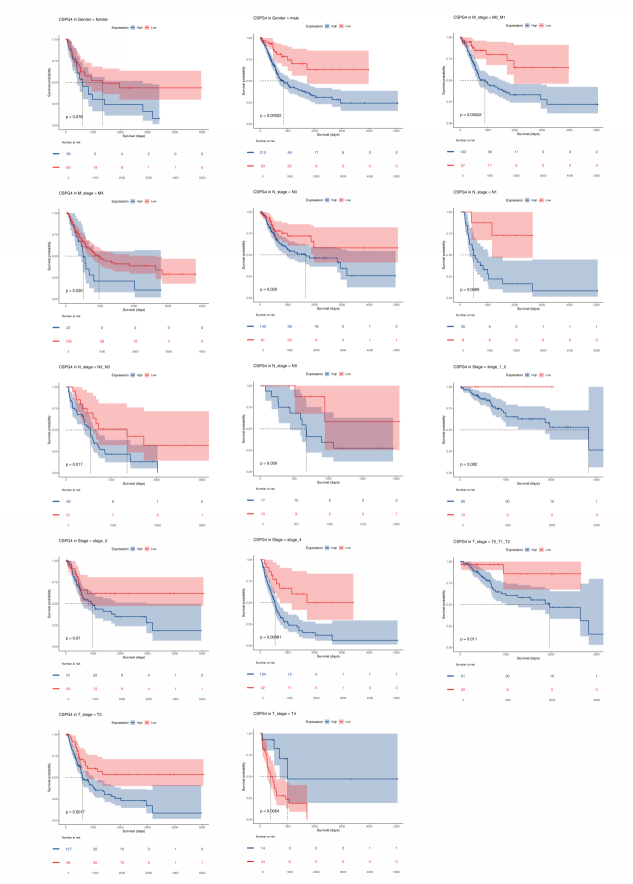
**

**Fig.S11: KM survival curves showing overall survival of patients in high and low expression sets of *CSPG4* in different clinical subgroups.**

**
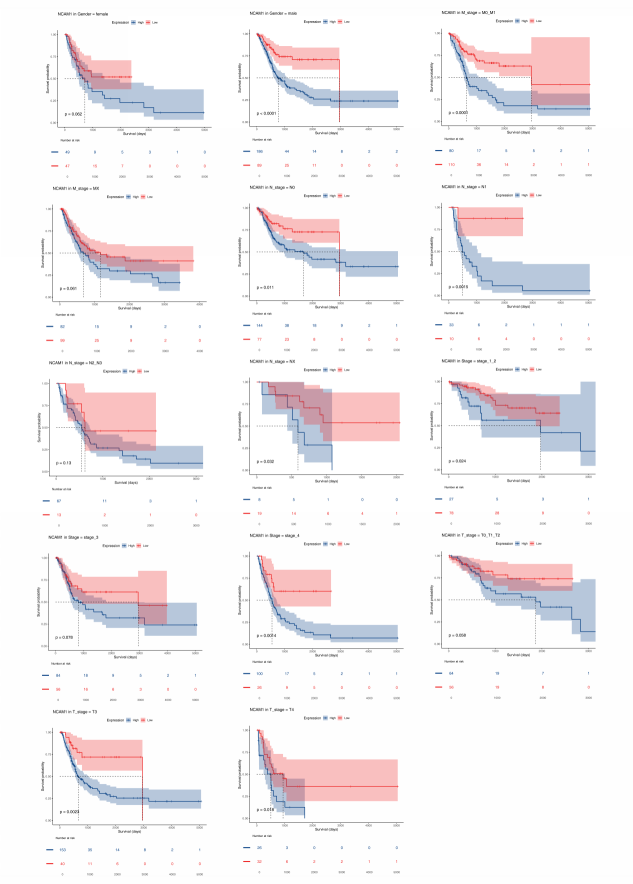
**

**Fig.S12: KM survival curves showing overall survival of patients in high and low expression sets of *NCAM1* in different clinical subgroups.**

**
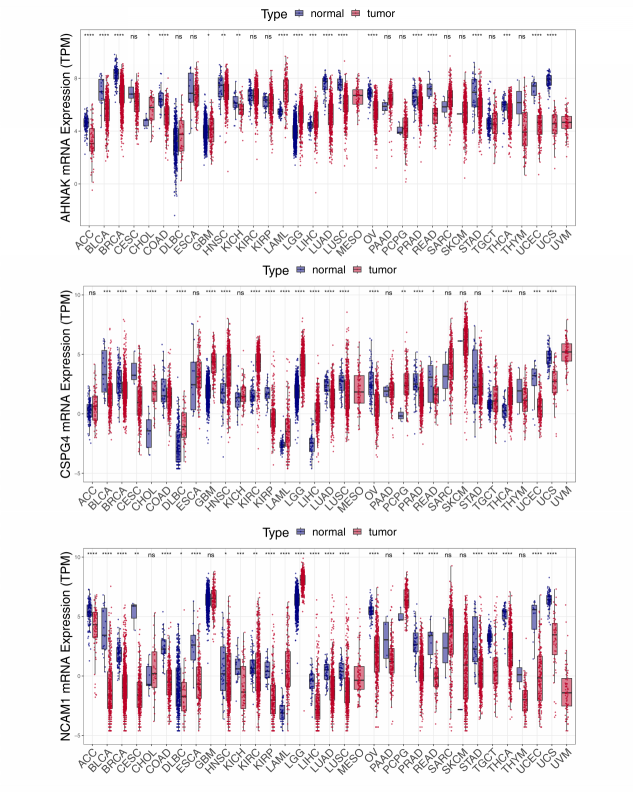
**

**Fig.S13: Boxplots of the expression of each key gene in the pan-cancer data**(Wilcoxon rank-sum test)**.** ns represented no significance, * represented p < 0.05, ** represented p < 0.01, *** represented p < 0.001, **** represented p < 0.0001.


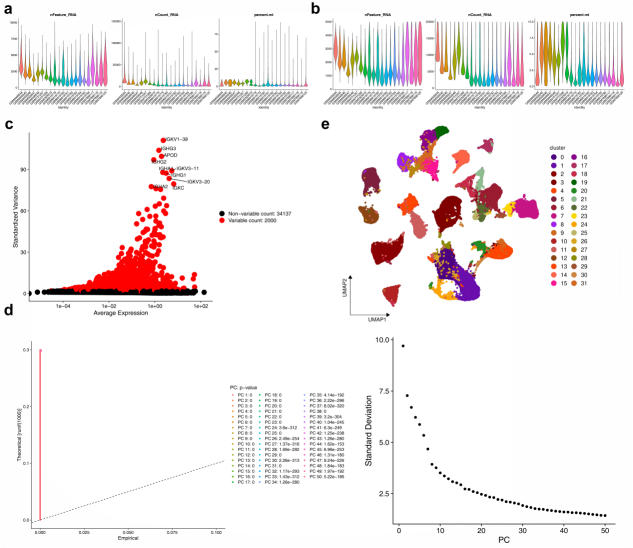


**Fig.S14: Single-cell quality control and dimensionality reduction clustering.**

**a, b** Quality control of single-cell data. **a** before, **b** after. **c** Top 2000 highly variable genes. **d** PCA dimensionality analysis. **e** The UMAP plot of 32 cell clusters.

**Supplementary Table Legends**

**Supplementary Table 1.** Clinical and pathological information of the 35 patients with bladder cancer included in the multi-omics analysis.

**Supplementary Table 2.** List of differentially expressed genes (DEGs) identified between bladder cancer and adjacent normal tissues by transcriptomic analysis.

**Supplementary Table 3.** Gene Ontology (GO) enrichment results for DEGs between bladder cancer and adjacent normal tissues.

**Supplementary Table 4.** Kyoto Encyclopedia of Genes and Genomes (KEGG) pathway enrichment results for DEGs between bladder cancer and adjacent normal tissues.

**Supplementary Table 5.** Somatic mutation gene (SMG) list identified by whole-exome sequencing (WES) in bladder cancer samples.

**Supplementary Table 6.** Differential abundance of microbial genera identified between bladder cancer and normal samples from intratumoral microbiome analysis.
